# Transient Host–Guest Complexation To Control Catalytic Activity

**DOI:** 10.1021/jacs.2c02695

**Published:** 2022-05-18

**Authors:** Michelle P. van der Helm, Guotai Li, Muhamad Hartono, Rienk Eelkema

**Affiliations:** Department of Chemical Engineering, Delft University of Technology, Van der Maasweg 9, 2629 HZ Delft, The Netherlands

## Abstract

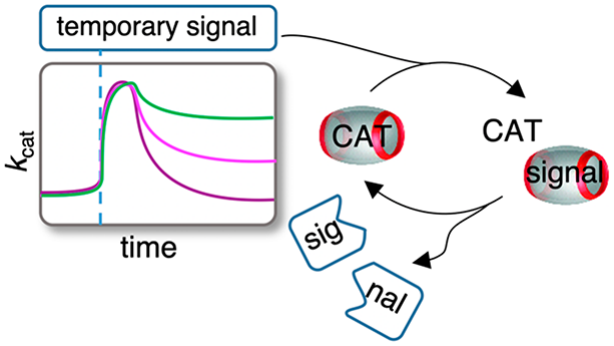

Signal transduction mechanisms are key to living systems. Cells respond to signals by changing catalytic activity of enzymes. This signal responsive catalysis is crucial in the regulation of (bio)chemical reaction networks (CRNs). Inspired by these networks, we report an artificial signal responsive system that shows signal-induced temporary catalyst activation. We use an unstable signal to temporarily activate an out of equilibrium CRN, generating transient host–guest complexes to control catalytic activity. Esters with favorable binding toward the cucurbit[7]uril (CB[7]) supramolecular host are used as temporary signals to form a transient complex with CB[7], replacing a CB[7]-bound guest. The esters are hydrolytically unstable, generating acids and alcohols, which do not bind to CB[7], leading to guest reuptake. We demonstrate the feasibility of the concept using signal-controlled temporary dye release and reuptake. The same signal controlled system was then used to tune the reaction rate of aniline catalyzed hydrazone formation. Varying the ester structure and concentration gave access to different catalyst liberation times and free catalyst concentration, regulating the overall reaction rate. With temporary signal controlled transient complex formation we can tune the kinetics of a second chemical reaction, in which the signal does not participate. This system shows promise for building more complex nonbiological networks, to ultimately arrive at signal transduction in organic materials.

## Introduction

Nature is full of elegant and complex signal transduction mechanisms to control key cellular processes. Cells respond to external and internal signals by altering enzymatic activity via covalent chemistry involving phosphorylation^1^ or noncovalently by allosteric activation or inhibition.^2,3^ Yet, artificial chemical reaction networks (CRNs) with similar complexity and control as found in living systems remain out of reach. Minimal signal integration in organic materials or reaction networks is uncommon.^4−7^ Incorporation of signal responsive catalysis in such systems is key to the regulation of artificial complex CRNs, reminiscent of their natural analogues.^8^ Some examples of signal-responsive catalysis have been reported for synthetic systems.^9−11^ A key feature of natural signal transduction is that the change in catalytic activity is temporary; i.e., over time the catalytic activity returns to a background level. This effect is achieved by depletion of the signal, or by active deactivation of the catalyst (e.g., by dephosphorylation). In contrast, in artificial systems, catalyst turn on is most often permanent, limiting potential for application.^12,13^ Here, we report a signal responsive catalyst that shows temporary catalytic activity. We achieve temporary activation with a tunable duration through the use of unstable signal molecules. Specifically, we use a combination of temporarily activated chemical reaction networks and host–guest chemistry to exert control over catalytic activity.

**Figure 1:**
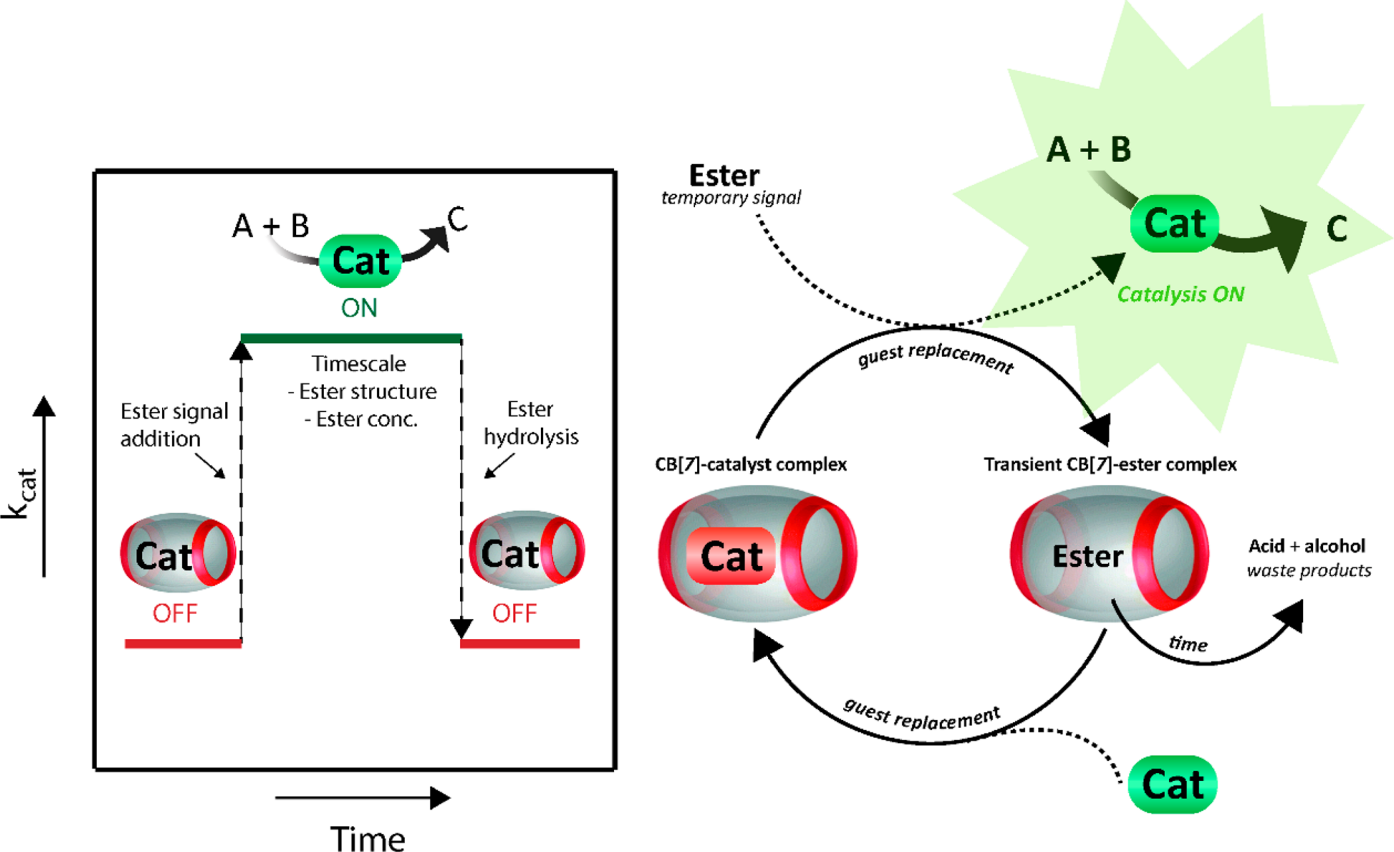
Transient complex formation of hydrolyzable ester signals with CB[7] to control the rate of a chemical reaction by catalyst capture and release. *k*_cat._ (represents the catalytic rate constant) versus time is shown, responding to ester addition and ester hydrolysis (left). Cat = catalyst.

The past decade witnessed a steep rise in the design of chemical reaction networks that are driven away from equilibrium by the conversion of a chemical fuel.^14−16^ There, a fuel molecule is used as a sacrificial reagent to drive an otherwise unfavorable chemical reaction, giving rise to nonequilibrium product distributions, transiently stable structures, and unusual system behaviors (i.e., oscillations, instabilities or chaotic behavior).^14,17−20^ Noncovalent interactions are frequently exploited to access transient structures, such as in the ATP-driven systems from Prins^21−24^ and in the multitude of transient supramolecular polymer systems assembled from (non)-biological building blocks.^17,25−29^ At the same time, host–guest chemistry has proven to be a powerful tool to regulate the reactivity of guest molecules, for example to control dissipative catalysis^30^ or fuel-driven transient crystallization.^31^ Here, in continuation of our previous work, we use cucurbit[7]uril (CB[7]) as a supramolecular host to encapsulate guest molecules in an aqueous environment.^9,10^ CB[7] has a high binding affinity for hydrophobic and positively charged molecules.^32^ We exploit this property to temporarily push a CRN away from equilibrium via transient complex formation with an unstable signal molecule that acts analogous to a chemical fuel. Inspired by the fuel-driven systems from the Walther group,^33^ we use various hydrolytically unstable esters as temporary signals to form a transient complex with CB[7]. The esters compete for CB[7] binding with the catalyst of the second chemical reaction, in this case aniline catalyzed hydrazone formation, and their hydrolysis controls the liberation of the catalyst from CB[7] and hence the catalytic activity and the overall reaction rate (Figure 1). First, we explain the design of the CRN and the choice of the temporary signals. Next, we demonstrate the proof-of-concept by controlling dye replacement. Finally, we show predictable control over the rate of the organocatalytic chemical reaction supported by a kinetic model.

## Results and Discussion

For the design of this CRN, the first requirement for the ester signals is a moderate to high binding affinity with CB[7]. Second, they should be hydrolyzable within the time scale of a catalytic chemical reaction. Third, the generated carboxylic acid and alcohol should have no binding affinity toward CB[7]. Hence, we considered glycine betaine esters as the ideal candidates and three different varieties were synthesized: methyl **1**, ethyl **2**, and isopropyl **3** (see Supporting Information (SI) for synthesis procedures). The positive charge from the ammonium gives favorable binding properties toward CB[7],^32^ resulting in binding constants (*K*_a_ in M^–1^) of order 10^4^–10^5^ (Table S1). Their binding inside CB[7] was also confirmed by NMR studies (SI Figures S3–S5). Amino acid esters are activated toward hydrolysis due to the neighboring α-amino group. Additionally, the positively charged betaine increases the hydrolysis rate compared to uncharged amino acid esters.^34^ Furthermore, the acid and alcohol hydrolysis products of the esters show no binding toward CB[7] (Table S1). The hydrolysis rates of the esters were measured at different pH and in the presence of CB[7] (SI Figures S6–S7). Resulting from electron-donating and steric effects, the hydrolysis rate displays the following order: methyl (fast) > ethyl > isopropyl (slow). Logically, a higher pH increases the ester hydrolysis rate and the presence of CB[7] slows down the hydrolysis. In the presence of CB[7] the hydrolysis of the isopropyl ester is even almost entirely switched off (SI Figure S7C,D).

**Figure 2:**
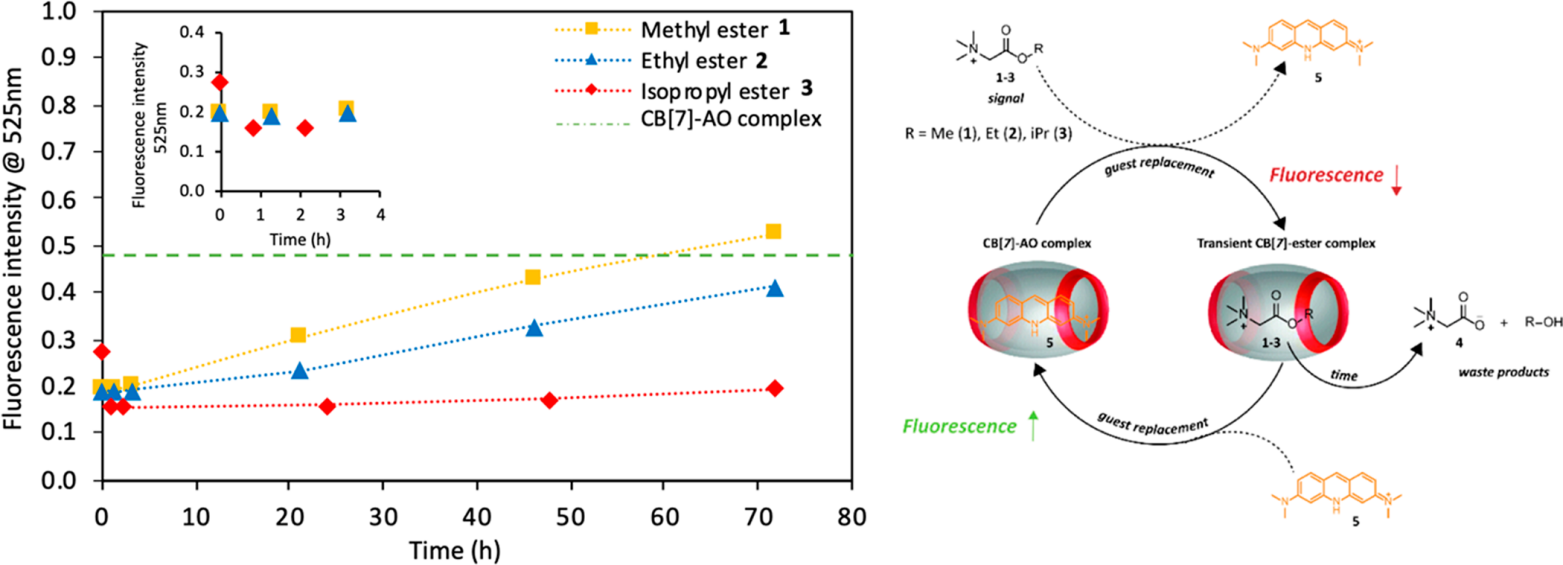
Fluorescence intensity of acridine orange (AO) **5** in and outside CB[7] over time at 525 nm (maximum in emission spectrum) with addition of different esters. The fluorescence intensity for **5** is high when inside CB[7] (green dashed line). When the esters replace the dye inside CB[7] the fluorescence intensity is lowered (from *t* = 0). The fluorescence intensity increases again when the esters hydrolyze over time and the dye is captured inside CB[7]. Conditions: esters (2.68 mM methyl **1**, 0.67 mM ethyl **2** or 0.13 mM isopropyl **3**) with 0.054 mM CB[7]and 0.027 mM AO 5 in sodium phosphate buffer 100 mM pH 7.5 at RT. Samples were excited at wavelength 465 nm. Dotted lines connecting the data are there to guide the eye.

We used a fluorescent dye replacement study as a proof-of-concept transient binding assessment (Figure 2). We use acridine orange (AO) **5**, which has a p*K*_a_ of 9.8 and is predominantly present in the protonated form at pH 7.5. The fluorescence intensity of the AO dye **5** is known to increase when bound inside CB[7].^35−37^ Initially, the fluorescence intensity of AO and CB[7] at 525 nm (maximum in emission spectrum) is about 0.5 (start in Figure 2 shown as green dashed line).

As is apparent from Figure 2, when the esters are added the fluorescence intensity drops, which indicates that the esters replace the dye inside CB[7]. The drop in fluorescence intensity happens instantaneously for methyl **1** and ethyl ester **2**. However, for isopropyl ester **3**, where because of the higher binding constant only a low concentration is needed to reach the same percentage of transient ester⊂CB[7] complex, the initial drop is more gradual (Figures 2, S11). Over time the esters hydrolyze, generating glycine betaine **4** and an alcohol as waste products. Due to the negative charge on **4** the binding affinity for CB[7] is lost and the dye is slowly captured inside the now vacant CB[7] again. This process is demonstrated by the increase in fluorescence over time in Figure 2. In accordance to the height of the hydrolysis rate constants for the different esters (SI Figures S6–S7), the fluorescence intensity increases more rapidly for methyl ester **1**, followed by ethyl ester **2**, and finally isopropyl ester **3**. Thus, the chemical structure of the ester signal has a direct and strong effect on the replacement rate. Overall, with this dye replacement experiment we illustrate that we can control the rate of a second process (i.e., dye capture and release) by controlling transient complex formation through a temporary signal.

**Figure 3:**
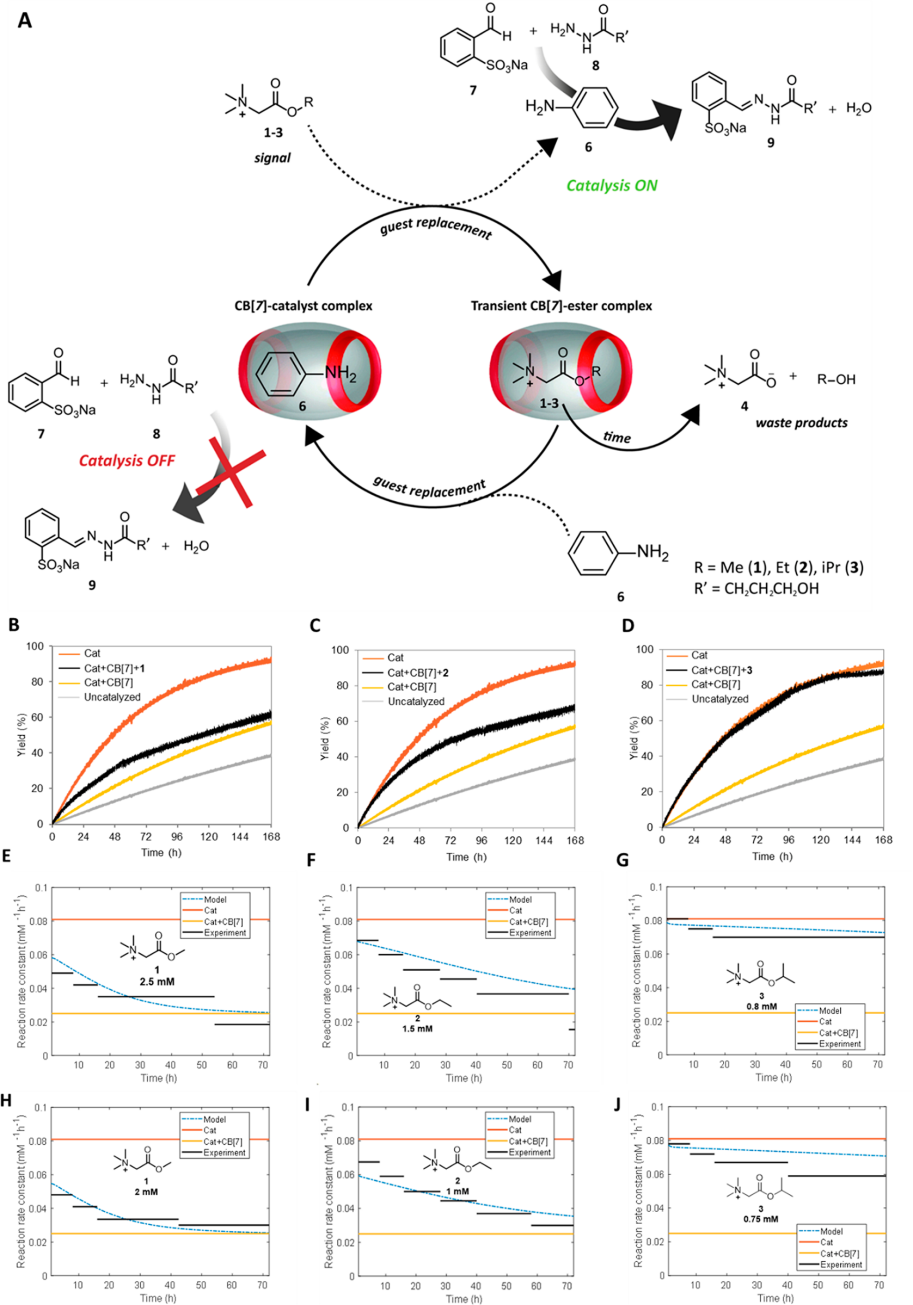
(A) Transient complex formation of hydrolyzable ester signals with CB[7] to control the rate of hydrazone formation by aniline catalyst 6 capture and release. Yield (B–D) and rate constant (E–J) for hydrazone formation as a function of time at various catalytic conditions. Values extracted from experimental data with ester signals (black lines) are compared with the model (blue dashed line): (B,E) Methyl ester 1 **2**.5 mM, (C,F) Ethyl ester 2 1.5 mM, (D,G) Isopropyl ester **3** 0.8 mM, (H) Methyl ester **1** 2 mM, (I) Ethyl ester **2** 1 mM and (J) Isopropyl ester **3** 0.75 mM. Conditions: pH 7.5, 100 mM phosphate buffer; 0.2 mM aldehyde 7, 0.02 mM hydrazide **8**, 0.2 mM catalyst **6** and 0.6 mM CB[7].

Taking this one step further, we use the same transient complex formation strategy to control catalytic activity in time. In our previous work we showed that CB[7] can be used to control aniline **6** catalyzed hydrazone formation in a buffered system.^10^ Here, we exploit the same reaction between aldehyde **7** and hydrazide **8** to form hydrazone **9** in combination with the hydrolyzable esters to achieve transient control over the reaction rate (Figure 3A). In order to measure the reaction rate and determine the rate constant, the yield of hydrazone product **9** is determined with UV–vis by following the absorbance at 287 nm (see SI Hydrazone UV–vis absorbance and reaction kinetics). As we confirmed previously, aniline **6** binds moderately strongly to CB[7] with a *K*_a_ of 2.78 × 10^4^ M^–1^ and the reactants and product do not bind to CB[7] (SI Figure S1 and Table S1). To illustrate the change in reaction rate, we calculated the reaction rate constant over time for the various conditions and different time intervals: with only catalyst; catalyst with CB[7] and catalyst with varying concentrations of esters **1**–**3** (Figure 3B–J and see SI Figures S13–S14 for the yield of hydrazone **9** and the slopes for the determination of the rate constants). With 0.2 mM catalyst **6** hydrazone formation is accelerated with a rate constant of 0.081 mM^–1^ h^–1^, whereas by addition of 0.6 mM CB[7] a rate decrease is observed with a rate constant of 0.025 mM^–1^ h^–1^. The reaction is not completely switched off due to a background reaction with constant 0.014 mM^–1^ h^–1^ (SI Figure S19) and with 0.6 mM CB[7] only 92% of catalyst is complexed with CB[7], together giving the observed nonzero rate constant. Upon addition of the ester signals a guest replacement takes place. The esters replace the catalyst inside CB[7], liberating the catalyst, which in turn starts to accelerate the hydrazone formation. However, over time the esters hydrolyze and the catalyst is gradually captured inside CB[7] again. This behavior is illustrated by a decrease in the reaction rate constant (Figure 3E–J black lines). For the ester experiments we calculated the reaction rate constant for various time intervals based on the changing slopes with linear curve fitting to the pseudo-first-order eq (SI Section 7.2). Because of data scatter and shallow slopes in the UV data, the time intervals had to remain large to guarantee good fits for accurate reaction rate constant determination (*R*^2^ > 0.9, Table S2). Especially at the end of the measurement time the very shallow slopes in the UV absorbance required the use of larger time intervals (SI Figure S15).

Overall, the ester structure and concentration control the decrease in reaction rate of hydrazone **9** formation. With 2.5 mM methyl ester **1** the catalysis is switched off after about 50 h (Figure 3B,E) with about 60% yield of hydrazone **9** after 168 h, whereas with [**1**] = 2 mM the switch off time reduces to 40 h (Figure 3H) with a similar yield of hydrazone **9** after 168 h (SI Figure S14A). For 1.5 mM and 1 mM ethyl ester **2** the switch off time is about 70 h (Figure 3F black line) and about 60 h (Figure 3I black line), respectively, both showing about a 68% yield of **9** after 168 h (SI Figure 3C, S14C). However, the isopropyl ester **3** acts as a near-permanent guest, as with both 0.8 and 0.75 mM the reaction rate hardly reduces over time (Figure 3G and 3J black lines), both concentrations of **3** giving about 90% yield of hydrazone **9** after 168 h (Figures 3D, S14E). The rate constant of the reaction changes over time due to a changing free catalyst concentration in solution as a consequence of the ester hydrolysis. Given these long reaction times, we also investigated side product formation (see SI section 9 Control experiments). NMR and LC-MS measurements of hydrazone formation reactions catalyzed by **6** in the presence of esters **1**–**3** confirm the generation of hydrazone **9** and hydrolysis of esters without significant side reactions (Figures S21–S23). Ester blank reactions with and without catalyst **6** (SI Figures S19–S20) show an increasing background reaction caused by increased carboxylic acid formation with higher ester concentration.^38^

Using the equilibrium relations of esters and catalyst with CB[7] and the previously determined ester hydrolysis rate constants in and outside CB[7], we designed a kinetic model to determine the concentration of all the species over time (see SI section 7 Kinetic model and Figures S16–S17). Based on the free catalyst concentration as a function of time, we calculate the changing rate constant for hydrazone formation over time and compare it with the experimental data. As is apparent from Figure 3B–G (blue dashed lines) the modeled rate constant describes the general trend shown by the experimental data accurately for varying ester structures and concentrations. Although there are quantitative differences between the model and the measured data, the decay profile of the rate constant is described qualitatively by the model within the limits set by the catalyzed and inhibited rate constant values (horizontal lines).

**Figure 4:**
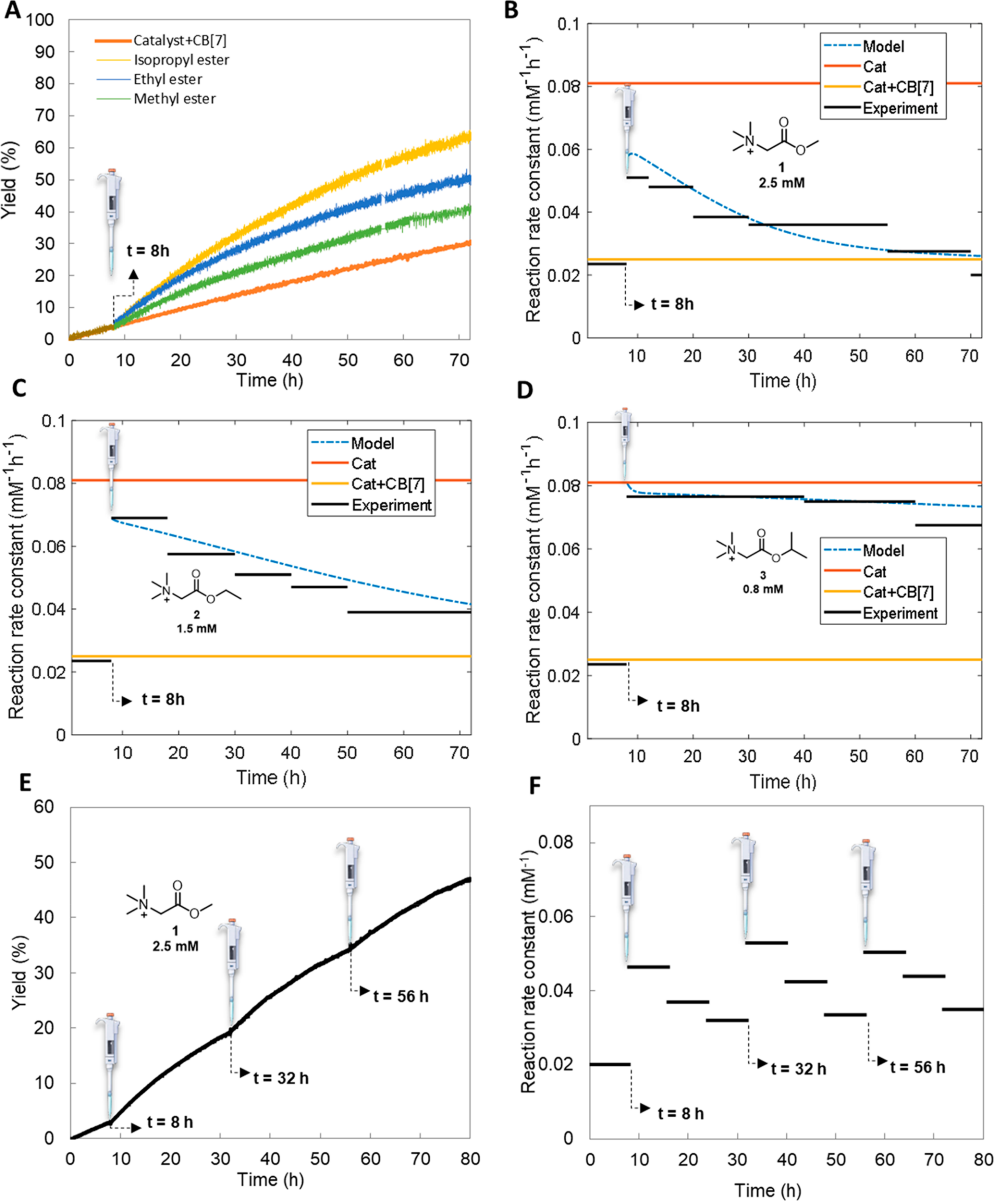
(A) Yield of hydrazone **9** for *in situ* addition of esters **1**–**3** at *t* = 8 h. Rate constant for hydrazone formation as a function of time at various catalytic conditions. Experimental data with ester signals (black lines) are compared with the model (blue dashed line): (B) Methyl ester **1** 2.5 mM, (C) Ethyl ester **2** 1.5 mM and (D) Isopropyl ester 3 0.8 mM. Conditions: pH 7.5 100 mM phosphate buffer, 0.2 mM aldehyde **7**, 0.02 mM hydrazide **8**, 0.2 mM catalyst **6** and 0.6 mM CB[7], (E) Yield of hydrazone **9** and (F) Rate constant for hydrazone formation as a function of time for 3 consecutive additions of methyl ester (1) at *t* = 8, 32, and 56 h. Conditions: pH 7.6 100 mM phosphate buffer, 2.5 mM methyl ester **1** (×3), 0.2 mM aldehyde 7, 0.02 mM hydrazide **8**, 0.2 mM catalyst **6**, and 0.42 mM CB[7].

Instead of adding the esters from the start, an *in situ* catalyst activation experiment was also performed (Figure 4). At the start, the rate of all experiments is similar to that of the catalyst⊂CB[7] experiment. Yet, when the ester signals are added after 8 h, they effectively replace the catalyst inside CB[7]. Hence, in Figure 4B–D after ester addition a rate increase is observed, indicating that catalyst **6** is liberated, accelerating the hydrazone formation. Next, upon ester hydrolysis a gradual decay in rate is observed. The decrease in rate constant and the final yield of hydrazone **9** follows a similar trend as in Figure 3 with the methyl ester **1** hydrolyzing the fastest (lowest yield of **9**) and the isopropyl ester **3** the slowest (highest yield of **9**). Also, for the *in situ* catalyst activation, the numerical model predicts the decrease in rate constant accurately but qualitatively for the different esters. For the methyl ester **1**, the model rate prediction is substantially higher from the start, possibly due to some ester hydrolysis already occurring. To further assess the stability of the system, we performed experiments with multiple ester additions (Figure 4E–F and SI Figure S18). Using methyl ester **1** as the signal, we could achieve at least 3 consecutive cycles of signal-controlled transient catalysis (Figure 4E–F). Sequential addition of ester **1** at *t* = 8, 32, and 56 h leads to temporal up–down regulation of the reaction rate. Noteworthy, a slightly higher pH (pH 7.6) and lower concentration of CB[7] (0.42 mM) have been adopted in this multiple addition experiment to ensure faster ester hydrolysis. These conditions were applied because, under the previous conditions (pH 7.5 and 0.6 mM CB[7]), the catalytic activity remained high after the third round of ester addition, most likely due to the accumulation of unhydrolyzed methyl ester **1** occupying the CB[7] cavity (Figure S18). In all, by transient complex formation with unstable ester signals inside a supramolecular host we have control over and can numerically predict the rate of a second chemical reaction by tuning the catalyst liberation time and hence the free catalyst concentration in solution.

## Conclusion

In this work, we used hydrolyzable esters as temporary signals to control catalytic activity in time. The system is based on an unstable signal molecule binding to a supramolecular host, leading to expulsion and activation of a catalyst bound in the host cavity. Decay of the signal molecule leads to reuptake and deactivation of the catalyst. As unstable signals, we used glycine betaine esters, with favorable binding toward CB[7]. Since the esters are unstable under aqueous conditions, they hydrolyze to form nonbinding acids and alcohols. With the transient ester CB[7] complexes, we demonstrated temporary dye release and reuptake, showing the feasibility of the concept. Next, we increased the network complexity and introduced a second chemical reaction. We used the temporary signal controlled transient complex formation to tune the reaction rate of aniline catalyzed hydrazone formation. Altering the ester structure and concentration gave different catalyst liberation times and changed the concentration of free catalyst and the overall reaction rate. The ester signals were effectively used for *in situ* activation of the organocatalyst and allow for repeated catalyst activation demonstrated by multiple signal additions. The experimental data were supported by a kinetic model. With transient complex formation we are able to control the kinetics of a second process, in which the signal itself does not take part (i.e., dye capture or chemical product formation). This generic nonequilibrium CRN, based on ester hydrolysis and supramolecular encapsulation, shows promise for building more complex nonbiological networks. The noncovalent (de)activation of catalysis could be applied to signal responsive soft materials, similar to the covalent (de)activation.^13^ Altogether, this work is a first step forward toward incorporation of signal transduction in artificial materials and chemical reaction networks.

## Supporting Information Available

Full synthetic procedure descriptions, characterization data, binding assays, catalysis experiments, and kinetic model (PDF)
