# Supplementary material for: Transient Host–Guest Complexation To Control Catalytic Activity
Source: J Am Chem Soc. 2022 May 18;144(21):9465–71. doi: 10.1021/jacs.2c02695 (PMC9164224; doi:10.1021/jacs.2c02695)
Supplement: Supplementary file 1 — ja2c02695_si_001.pdf [file ja2c02695_si_001.pdf]

Supporting information

Transient host-guest complexation to control catalytic activity

Michelle P. van der Helm[1], Guotai Li[1], Muhamad Hartono[1] and Rienk Eelkema*,[1]
[1] Department of Chemical Engineering, Delft University of Technology,
Van der Maasweg 9, 2629 HZ Delft, The Netherlands

***Corresponding Author:** Rienk Eelkema
Tel: +31 (0)15 27 81035; Email: R.Eelkema@tudelft.nl.
Department of Chemical Engineering, Faculty of Applied Sciences, Delft University of Technology,
Van der Maasweg 9, 2629 HZ Delft, The Netherlands

Table of Contents

1 Experimental details ... 2
- 1.1 General materials and methods ... 2
- 1.2 Glycine betaine esters (methyl 1, ethyl 2 and isopropyl 3) synthesis ... 2
- 1.3 Hydrazone product 9 synthesis ... 3
- 1.4 ITC binding constant measurement ... 3
- 1.5 Fluorescence assay of acridine orange 5 and CB[*7*] ... 3
- 1.6 UV-Vis assay to follow the hydrazone formation reaction ... 3

2 ITC binding constants with CB[7] ... 4
- 2.1 Catalyst, product and reactants ... 4
- 2.2 Esters and acid waste ... 5
- 2.3 Overview of CB[7] binding constants from ITC ... 6

3 Ester-CB[7] binding ^{1}H NMR spectra ... 7

4 Ester hydrolysis profiles ... 9

5 Dye CB[*7*] fluorescence spectra ... 14

6 Hydrazone UV-VIS absorbance ... 15

7 Kinetic model ... 16
- 7.1 Ester hydrolysis reaction kinetics ... 16
- 7.2 Hydrazone formation reaction kinetics ... 16
- 7.3 Equilibrium concentration calculation of dye/catalyst/ester⊂CB[7] complex ... 17
- 7.4 Matlab numerical model of differential equations ... 18

8 Hydrazone formation reactions ... 20

9 Control experiments ... 28
- 9.1 Ester blank reactions ... 28
- 9.2 NMR and MS controls ... 30

10 NMR and MS spectra ... 32

References ... 38

1 Experimental details

1.1 General materials and methods

Chemicals were purchased in the highest purity and used without further purification unless reported otherwise. Tetrahydrofuran (THF), methanol and diethyl ether (DE) of technical grades were purchased from VWR International. Acridine orange **5** (AO) hydrochloride solution (10 mg/mL) and aniline catalyst **6** (99.5%) of ACS reagent grade were purchased from Sigma Aldrich. CB[7] hydrate was purchased from Strem Chemicals Inc, and based on ITC measurements it was estimated to contain about 30 wt% hydration water. *o*-Sulfobenzaldehyde **7** was purchased from Honeywell Fluka Fischer Scientific and *p*-hydroxybutiric acid hydrazide **8** (98%) from Alfa Aesar. Methyl bromoacetate (99%), ethyl bromoacetate (98%), isopropyl bromoacetate (99%) and trimethylamine solution (31-35 wt% ethanol) were purchased from Sigma Aldrich. Solid salts were used for the preparation of aqueous buffers. Sodium phosphate monobasic monohydrate (98%) was purchased from Sigma Aldrich, while sodium phosphate dibasic (+99%, analysis grade) was from Acros Organics. Unless stated otherwise, all preparations and analyses were performed at room temperature (RT) (~21 °C) and atmospheric pressure. Nuclear Magnetic Resonance (NMR) experiments were performed using Agilent-400 MR DD2 (400 MHz for ^{1}H and 100.5 MHz for ^{13}C) at 25 °C using residual deuterated solvent signals as internal standard. To suppress the water peak, PRESAT configuration (suppress one highest peak) was used. UV-Vis spectroscopic experiments were carried out using Analytik Jena Specord 250 spectrophotometer; quartz cuvette with a 1 cm path length, volume of 3 mL, at a controlled temperature of 25 °C. Fluorescence spectra were recorded on a JASCO J-815 CD spectrometer (sensitivity 450 Volts, data pitch 1 nm, band width 5 nm, excitation wavelength of 465 nm for AO **5**); black quartz cuvette with a 1 cm path length, volume of 200 µL, at RT. Isothermal titration calorimetry (ITC) experiments were performed using a MicroCal VP-ITC. Liquid Chromatography–Mass Spectrometry (LC-MS) was performed on a Shimadzu Liquid Chromatograph Mass Spectrometer 2010, LC-8A pump with a diode array detector SPD-M20. Negative and/ or positive mode Electro Spray Ionization Mass Spectrometry (ESI-MS) was used for the peak assignment.

1.2 Glycine betaine esters (methyl 1, ethyl 2 and isopropyl 3) synthesis

Following the literature [1], for methyl ester **1**, trimethylamine (1.5 equivalents; 33 wt % in EtOH) was added to a solution of methyl bromoacetate (1 equivalent) in THF (50 mL/g). The reaction mixture was stirred for 24h at RT, during this time the product precipitated as a white solid. The resulting suspension was cooled with ice, filtered, washed with ice-cold Et_2O and dried in the vacuum oven at 40 °C overnight to yield the ammonium salt with bromine counter ion as white solid. For the ethyl and isopropyl esters (**2-3**) the same procedure was followed with ethyl bromoacetate or isopropyl bromoacetate respectively.

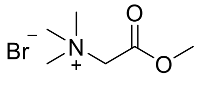

1

Methyl ester **1** (yield 95%, 1.5 g, 7.1 mmol): **^{1}H NMR** (400 MHz, D_2O): δ = 4.37 (s, 2H, $-CH_2-$), 3.87 (s, 3H, $-CH_3$), 3.36 (s, 9H, $-CH_3$). **^{13}C NMR** (100.5 MHz, D_2O): 165.7, 63.4, 54.0, 53.2. **MS** (ESI, positive) m/z: 132 [$(M)^+$] (expected m/z = 132.10).

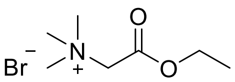

2

Ethyl ester **2** (yield 97%, 1.5 g, 6.6 mmol): **^{1}H NMR** (400 MHz, D_2O): δ = 4.29 (s, 2H, $-CH_2-$), 4.32-4.26 (q, 2H, $-CH_2-$, *J* = 7 Hz), 3.31 (s, 9H, $-CH_3$), 1.29-1.26 (t, 3H, -CH_3, *J* = 7 Hz). **^{13}C NMR** (100.5 MHz, D_2O): 165.2, 63.6, 63.3, 54.0, 13.1. **MS** (ESI, positive) m/z: 146 [$(M)^+$] (expected m/z = 146.12).

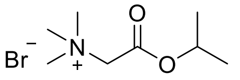

3

Isopropyl ester **3** (yield 99.5%, 1.2 g, 5.0 mmol): **^{1}H NMR** (400 MHz, D_2O): δ = 5.21-5.15 (m, 1H, -CH-), 4.31 (s, 2H, $-CH_2-$), 3.35 (s, 9H, $-CH_3$), 1.34-1.32 (d, 6H, $-CH_3$, *J* = 7 Hz). **^{13}C NMR** (100.5 MHz, D_2O): 164.6, 72.2, 63.8, 54.0, 20.6. **MS** (ESI, positive) m/z: 160 [$(M)^+$] (expected m/z = 160.13).

1.3 Hydrazone product 9 synthesis

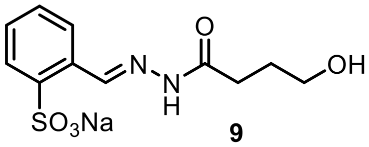

9

The synthesis was performed according to our previous reported procedure [2]. The purity of the product was confirmed by NMR and MS: N.B. Extra splitting of the peaks in the NMR spectrum is due to *cis* and *trans* isomers. **^{1}H NMR** (400 MHz, D_2O): δ = 8.96 (s, 1H, -CH=N-), 8.15-8.13 (d, 2H, Ar-H, *J* = 7 Hz), 7.96-7.94 (d, 2H, Ar-H, *J* = 7 Hz), 7.68-7.59 (m, 2H, Ar-H), 3.71-3.65 (t, 2H, $-CH_2-$, *J* = 7 Hz), 2.49-2.45 (t, 2H, $-CH_2-$, *J* = 7 Hz), 1.97-1.90 (m, 2H, $-CH_2-$). **^{13}C NMR** (100.5 MHz, D_2O): 173.0, 147.5, 141.4, 131.7, 130.7, 130.1, 127.3, 126.9, 60.7, 30.6, 27.4. **MS** (ESI, negative) m/z: 285 [$(M-Na^+)^-$] (expected m/z = 285.05). **Extinction coefficient** in sodium phosphate buffer (100 mM, pH 7.5) at 287 nm: 19.2 $mM^{-1}cm^{-1}$.

1.4 ITC binding constant measurement

A solution of the guest molecule ($3x10^{-5}$ mol from 10 mM stock) was titrated to CB[7] (0.35 mM) solution at 25 °C. Unless stated differently, the solutions were prepared in sodium phosphate buffer 100 mM pH 7.5. The first titration point out of 28 injection points was discarded. Binding constants were fitted with Microcal LLC ITC Origin 7 software.

1.5 Fluorescence assay of acridine orange 5 and CB[*7*]

Fluorescence measurements were performed in 100 mM sodium phosphate buffer pH 7.5 with 0.027 mM AO **5**, 0.054 mM CB[7] and esters (2.68 mM methyl **1**, 0.67 mM ethyl **2** and 0.13 mM isopropyl **3**) in black quartz cuvettes, path length of 1 cm (total reaction volume of 200 µL) at RT. Based on these concentrations, 96% of the CB[7] will be occupied by methyl ester **1**, and 96% by ethyl ester **2** and 97% by isopropyl ester **3**. The stock solutions were added in the following order: phosphate buffer, dye **5** and CB[*7*] (premixed for 1 h) and ester **1**-**3** solution. Teflon caps were used to close the cuvette. The cuvette was turned upside down to mix the solution. Samples were excited at wavelength 465 nm. N.B., AO **5** has a pK_a of 9.8 and is predominantly present in the protonated form at pH 7.5 [3].

1.6 UV-Vis assay to follow the hydrazone formation reaction

Unless stated otherwise, the hydrazone reaction was performed in 100 mM sodium phosphate buffer pH 7.5, containing 0.2 mM aldehyde **7**, 0.02 mM hydrazide **8**, 0.2 mM aniline **6**, and 0.6 mM CB[7] in quartz cuvettes, path length of 1 cm (total reaction volume of 3 mL) at 25 °C. The stock solutions of the reactants were added in the following order: catalyst **6** solution and CB[*7*] (premixed for 1 h), aldehyde **7** solution, phosphate buffer and hydrazide **8** solution. Teflon caps were used to close the cuvette. The cuvette was turned upside down five times to mix the solution. The product peak was followed (at 287 nm) using a 6-sample holder (configuration: slow time scan, scan every 20 s). The pH was measured before and after the reaction. Experiments with esters **1**-**3** (2.5/ 2 mM methyl **1**, 1.5/ 1 mM ethyl **2** and 0.8/ 0.75 mM isopropyl **3**) were performed similarly, with the ester **1**-**3** stock solution being added at the last moment. Based on these concentrations, 95%/ 94% of the methyl ester **1** will bind to CB[7], 97%/ 95% of ethyl ester **2** and 99%/ 99% of isopropyl ester **3**. The concentration of hydrazone product **9** was calculated with the extinction coefficient and Lambert-Beer law.

2 ITC binding constants with CB[7]

2.1 Catalyst, product and reactants

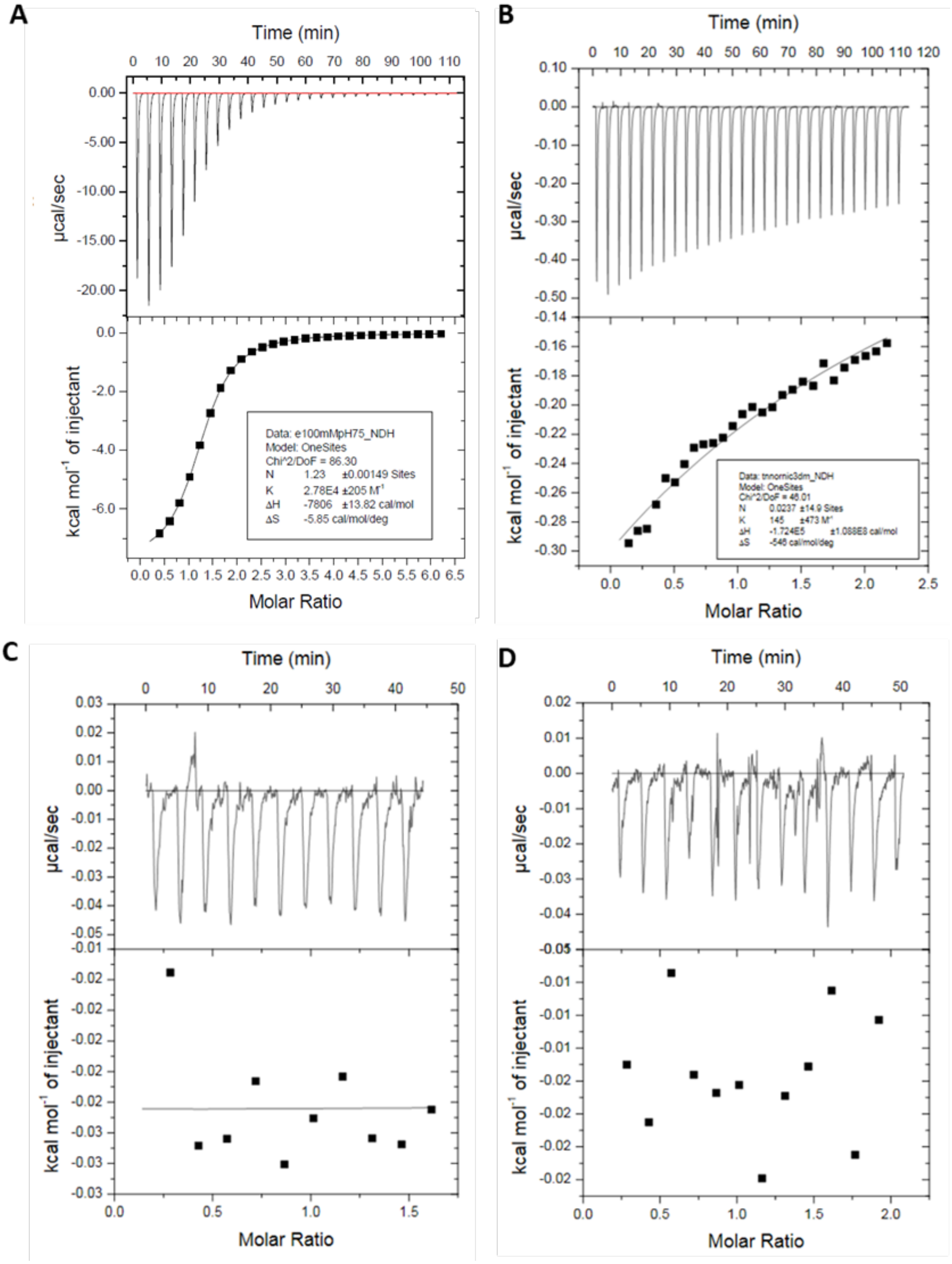

Figure S1: Binding constant measurement using ITC, guest (10 mM) and CB[*7*] (0.35 mM) in sodium phosphate buffer (pH 7.4, 100 mM) at RT. **(A)** Aniline catalyst **6**, **(B)** Hydrazone **9**, **(C)** Aldehyde **7** and **(D)** Hydrazide **8**.

2.2 Esters and acid waste

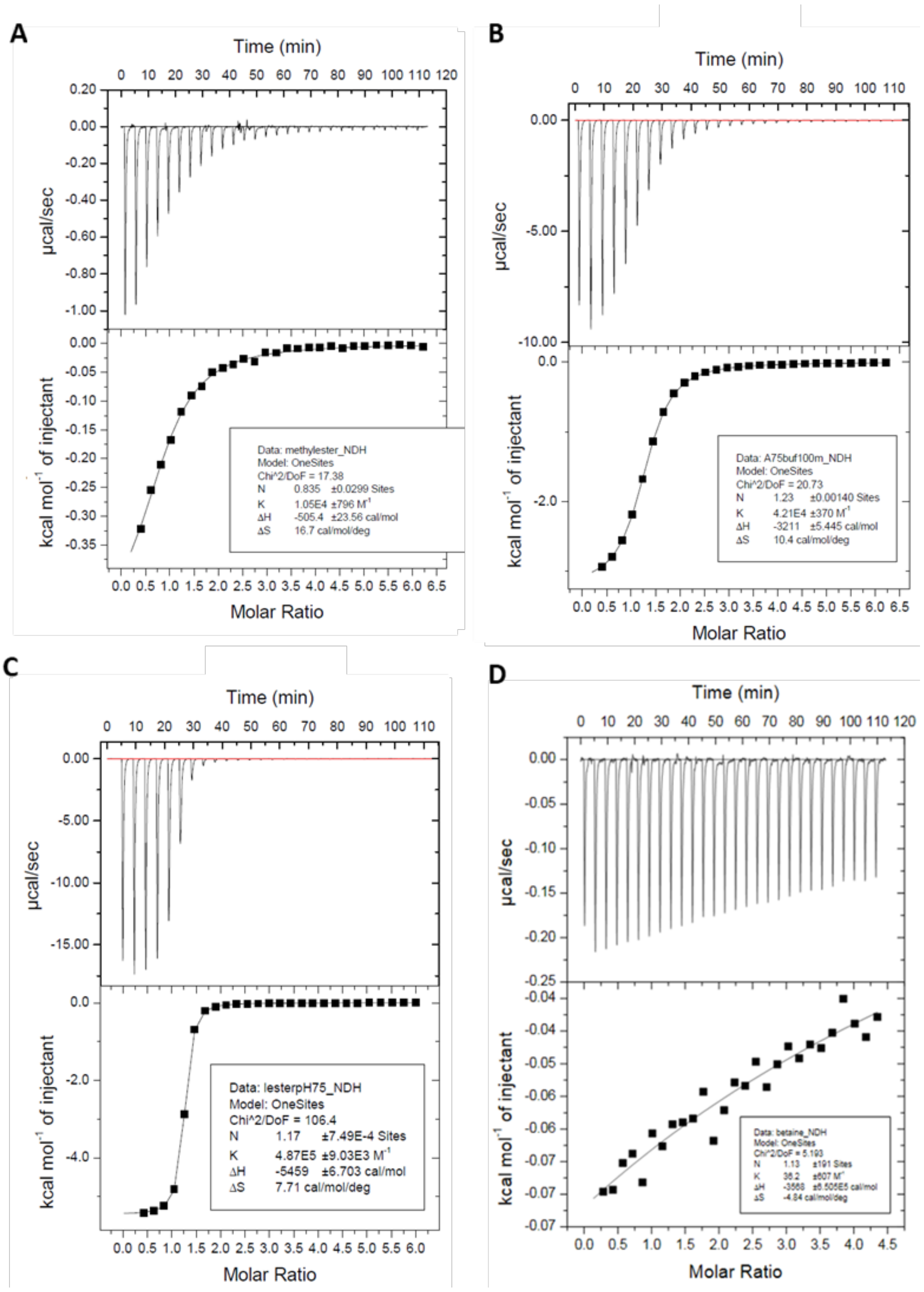

Figure S2: Binding constant measurement using ITC, esters or acid (10 mM) and CB[*7*] (0.35 mM) in sodium phosphate buffer (pH 7.5, 100 mM) at RT. **(A)** Methyl ester **1**, **(B)** Ethyl ester **2**, **(C)** Isopropyl ester **3** and **(D)** Betaine **4**.

2.3 Overview of CB[7] binding constants from ITC

Table S1: Binding constants (K_a) and thermodynamic binding values (ΔH and TΔS) for esters **1-3**, their hydrolysis products, acridine orange (AO) **5**, aniline catalyst **6**, aldehyde **7**, hydrazide **8** and hydrazine **9** as determined by Isothermal titration calorimetry (ITC) or from the literature. A negative ΔH value indicates an enthalpic gain, while a positive TΔS indicates an entropic gain. Not measurable means that the binding constant cannot be measured, because it is under the detection limit of the ITC. [1] The binding constant for AO **5** was taken from the literature obtained with fluorescence titration.

Molecular structure	Name	K_a, M^{-1}	ΔH, kcal mol^{-1}	TΔS, kcal mol^{-1}
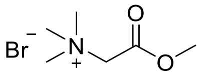 **1**	Glycine betaine methyl ester **1**	(1.05 ± 0.08) x 10^4	-0.505 ± 0.023	4.97
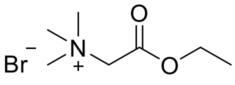 **2**	Glycine betaine ethyl ester **2**	(4.21 ± 0.037) x 10^4	-3.21 ± 0.05	3.01
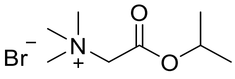 **3**	Glycine betaine isopropyl ester **3**	(4.87 ± 0.09) x 10^5	-5.46 ± 0.007	2.30
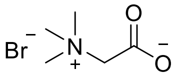 **4**	Glycine betaine **4**: Hydrolysis product of **1-3**	Not measurable	Not measurable	Not measurable
R−OH R= CH_3, CH_2CH_3, $CH(CH_3)_2$	Methanol/ ethanol/ isopropanol: Hydrolysis products of **1-3**	Not measurable	Not measurable	Not measurable
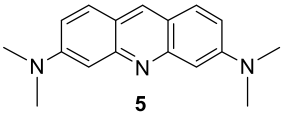	Acridine orange (AO) dye **5**	5.6 x 10^5[4] (pH 7.5 phosphate buffer saline)[1]	Not reported	Not reported
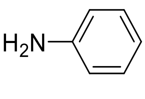 **6**	Aniline **6** (hydrazone formation catalyst)	(2.78 ± 0.21) x 10^4	-7.8 ± 0.014	1.7
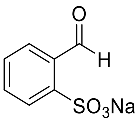 **7**	Aldehyde **7**	Not measurable	Not measurable	Not measurable
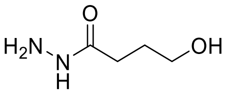 **8**	Hydrazide **8**	Not measurable	Not measurable	Not measurable
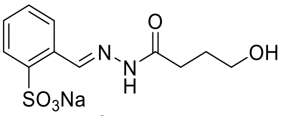 **9**	Hydrazone product **9**	Not measurable	Not measurable	Not measurable

3 Ester-CB[7] binding ^{1}H NMR spectra

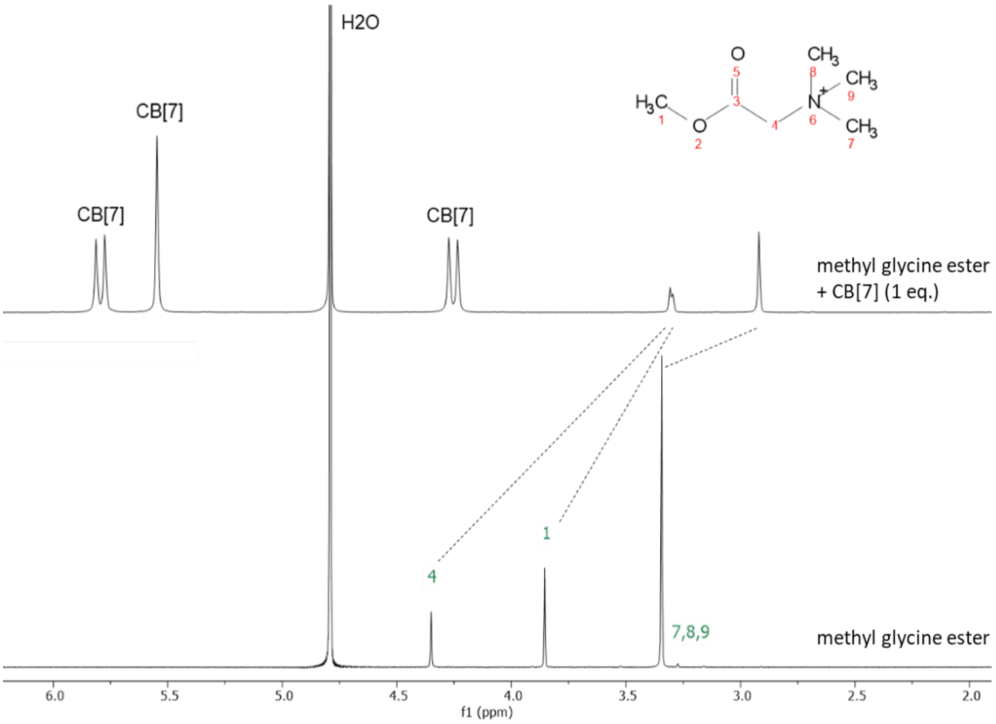

Figure S3: ^{1}H NMR spectra of methyl ester **1** (2 mM) in the absence and presence of 1 eq. of CB[7] in sodium phosphate buffer 100 mM, pH 7.5 at RT (NMR solvent D_2O).

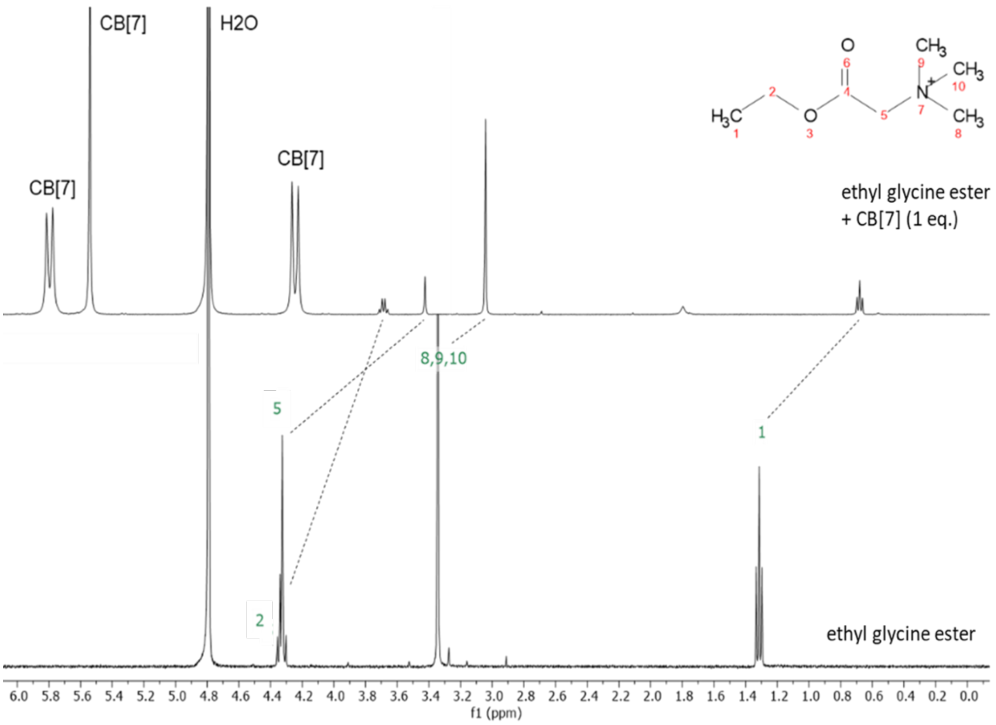

Figure S4: ^{1}H NMR spectra of ethyl ester **2** (2 mM) in the absence and presence of 1 eq. of CB[7] in sodium phosphate buffer 100 mM, pH 7.5 at RT (NMR solvent D_2O).

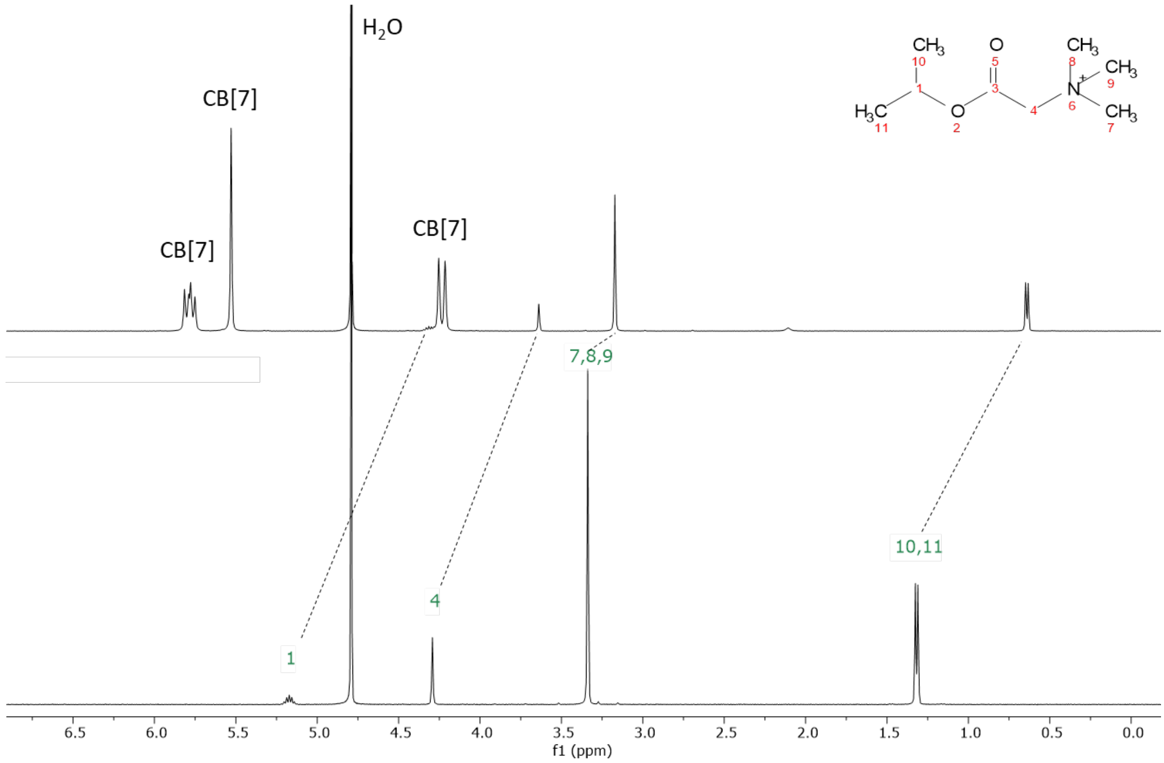

Figure S5: ^{1}H NMR spectra of isopropyl ester **3** (2 mM) in the absence and presence of 1 eq. of CB[7] in sodium phosphate buffer 100 mM, pH 7.5 at RT (NMR solvent D_2O).

4 Ester hydrolysis profiles

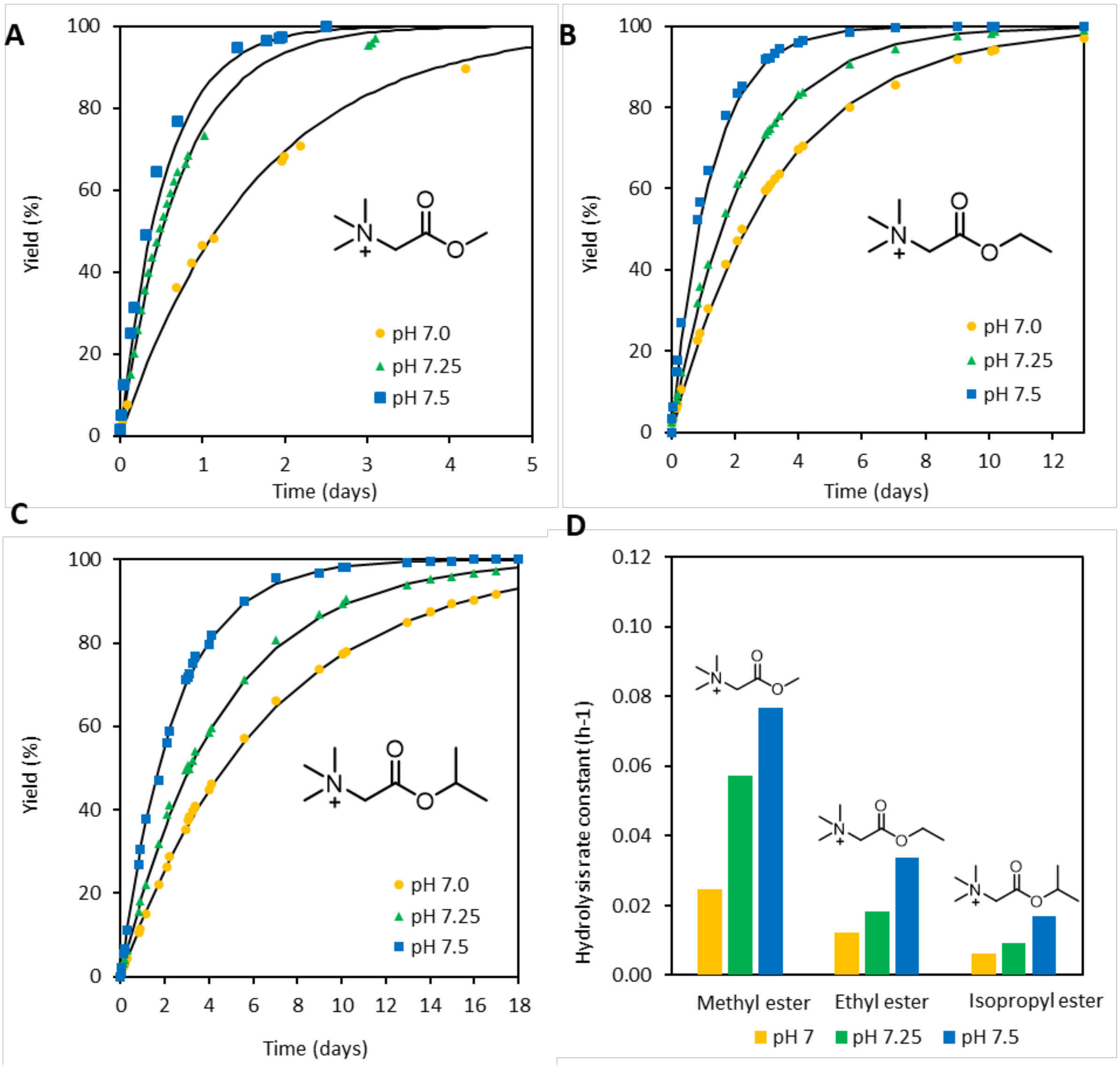

Figure S6: Ester hydrolysis profiles at different pH as determined by ^{1}H NMR. **(A)** Methyl ester **1**, **(B)** Ethyl ester **2**, **(C)** Isopropyl ester **3** and **(D)** A summary of the corresponding hydrolysis rate constants. Conditions: ester (2 mM) in sodium phosphate buffer 100 mM (pH 7.0, 7.25 or 7.5) at RT. The blue, green and yellow symbols are the experimental data points as determined by ^{1}H NMR. The black line shows a first-order reaction rate model based on the reaction rate constants shown in panel **(D)**.

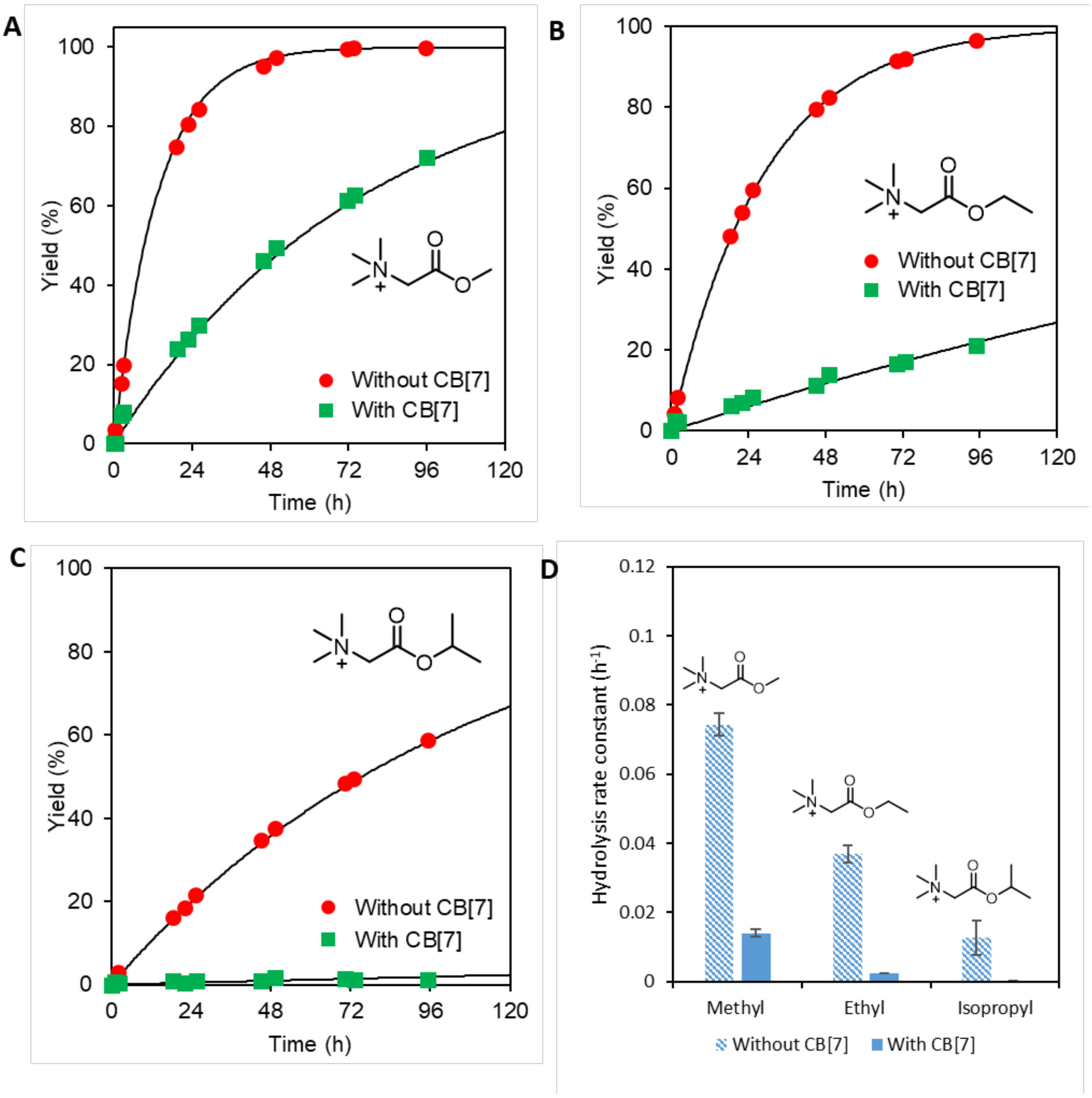

Figure S7: Ester hydrolysis profiles in the presence and absence of CB[7] as determined by ^{1}H NMR. **(A)** Methyl ester **1**, **(B)** Ethyl ester **2**, **(C)** Isopropyl ester **3** and **(D)** A summary of the corresponding hydrolysis rate constants (n=2). Conditions: esters (1 mM) (with 1.4 mM CB[7] for ethyl and isopropyl ester and 3 mM CB[7] for the methyl ester) in sodium phosphate buffer 100 mM pH 7.5 at RT. The red and green symbols are the experimental data points as determined by ^{1}H NMR. The black line shows a first-order reaction rate model based on the rate constants shown in panel **(D)**.

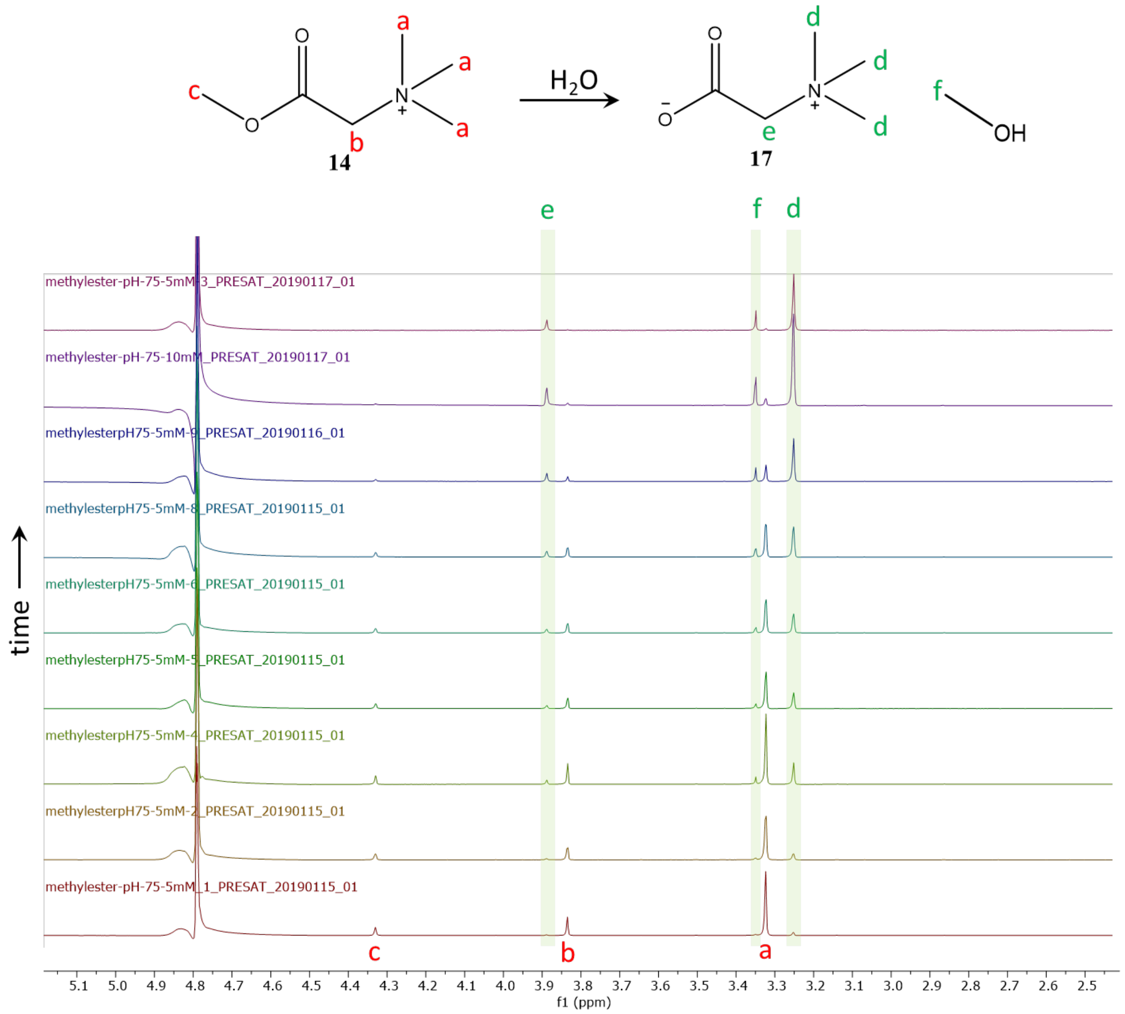

Figure S8: Example of ^{1}H NMR spectral analysis of the hydrolysis of methyl ester **1** in sodium phosphate buffer (100 mM, pH 7.5 with NMR solvent D_2O) at RT at different time intervals. The peaks corresponding to the ester (red) vanish over time, meanwhile the product peaks (green) intensify.

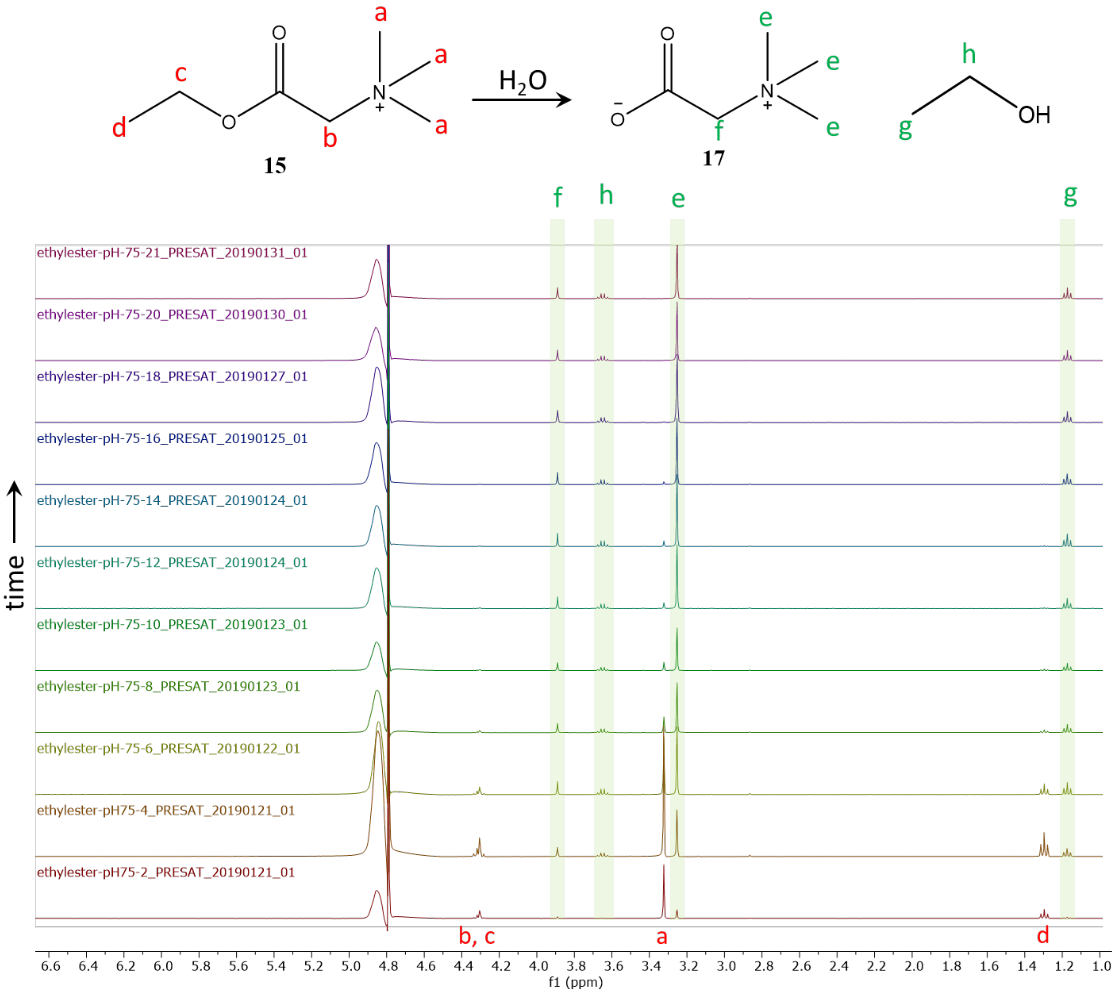

Figure S9: Example of ^{1}H NMR spectral analysis of the hydrolysis of ethyl ester **2** in sodium phosphate buffer (100 mM, pH 7.5 with NMR solvent D_2O) at RT at different time intervals. The peaks corresponding to the ester (red) vanish over time, meanwhile the product peaks (green) intensify.

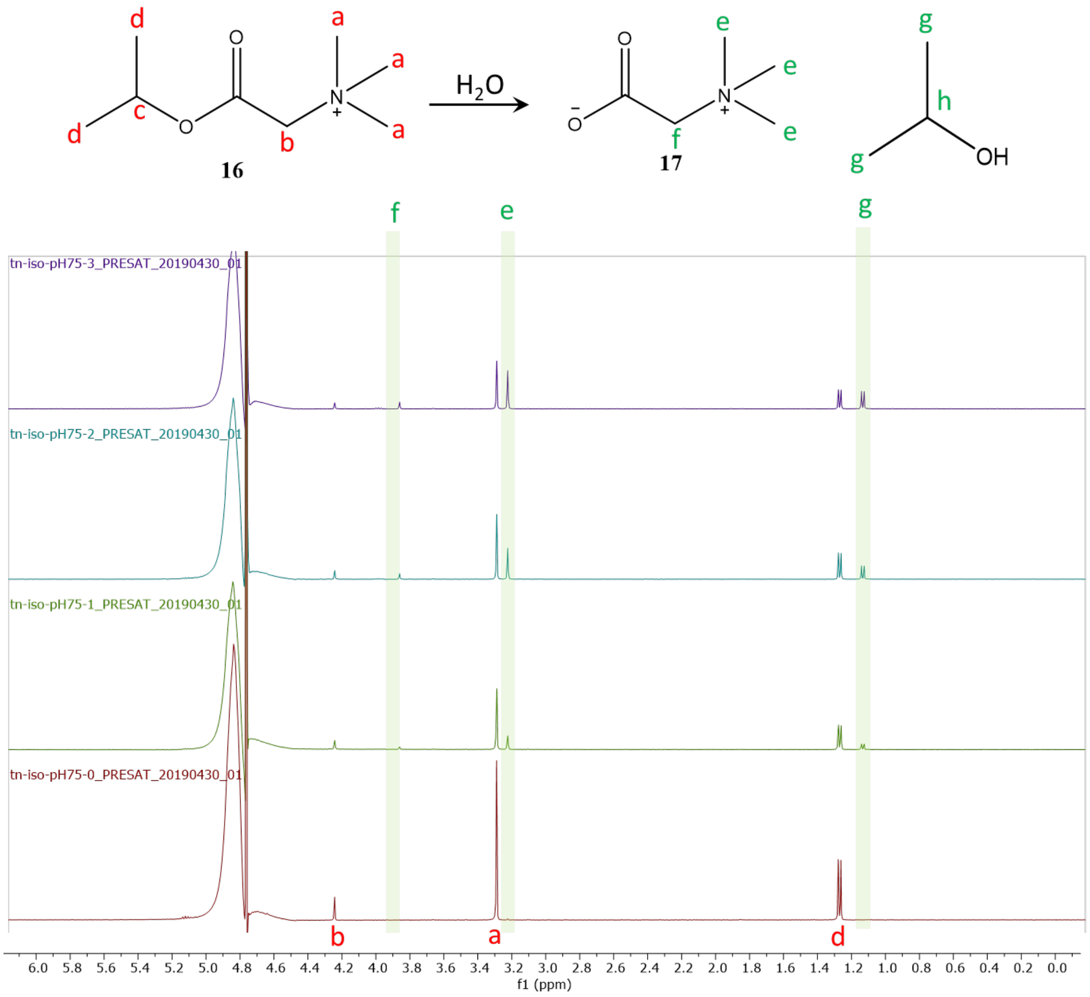

Figure S10: Example of ^{1}H NMR spectral analysis of the hydrolysis of isoproyl ester **3** in sodium phosphate buffer (100 mM, pH 7.5 with NMR solvent D_2O) at RT at different time intervals. The peaks corresponding to the ester (red) vanish over time, meanwhile the product peaks (green) intensify.

5 Dye CB[*7*] fluorescence spectra

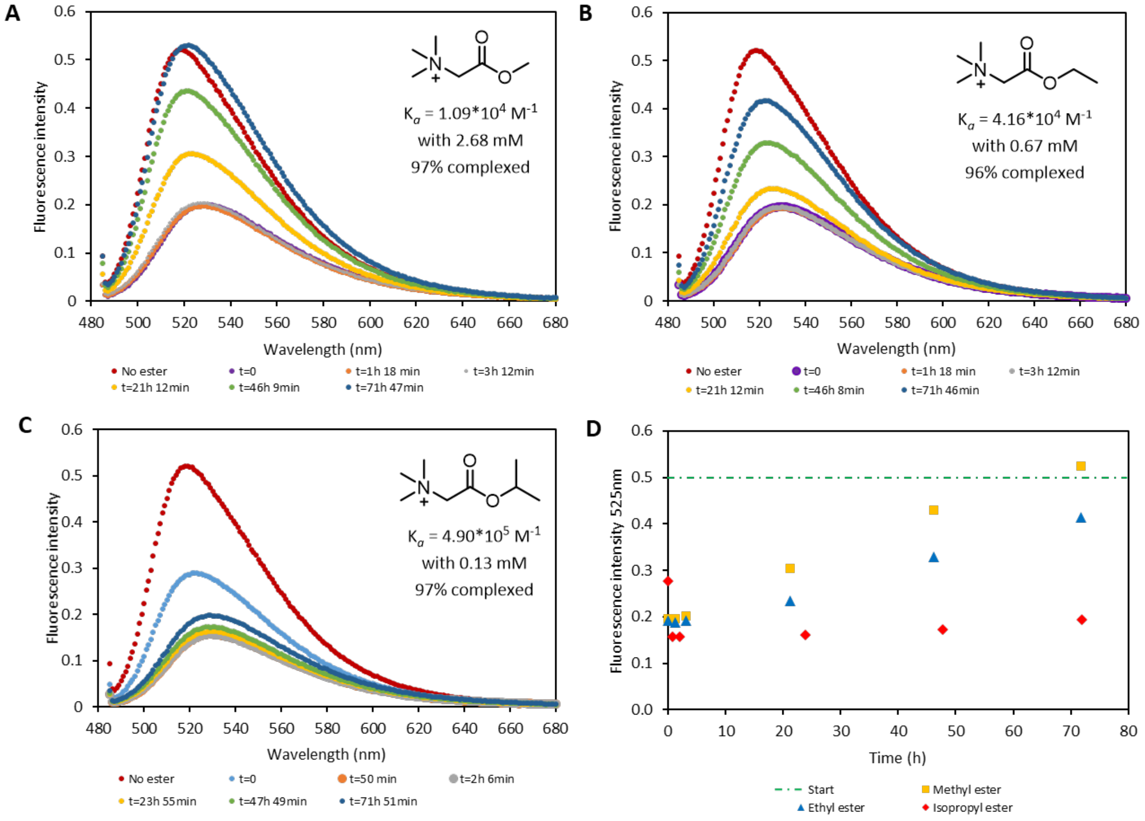

Figure S11: Acridine orange (AO) **5** fluorescence spectra over time in the presence of CB[7] and different esters. **(A)** Methyl ester **1**, **(B)** Ethyl ester **2**, **(C)** Isopropyl ester **3** and **(D)** Fluorescence intensity of AO **5** in and outside CB[7] over time at 525 nm (maximum in emission spectrum) with addition of different esters. Conditions: esters (methyl **1** 2.68 mM, ethyl **2** 0.67 mM and isopropyl **3** 0.13 mM) with 0.054 mM CB[7]and 0.027 mM AO **5** in sodium phosphate buffer 100 mM pH 7.5 at RT. Samples were excited at wavelength 465 nm.

6 Hydrazone UV-VIS absorbance

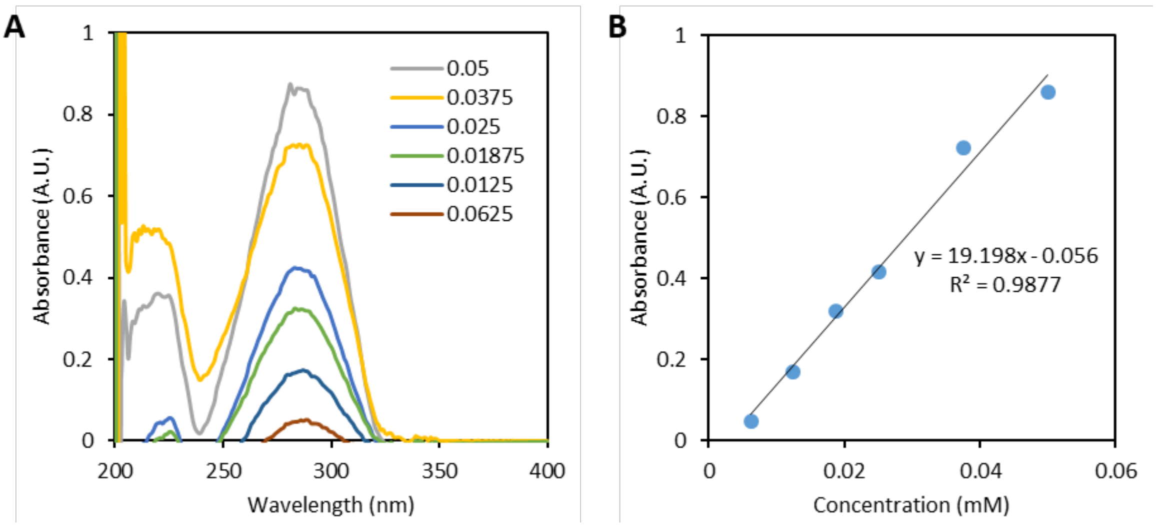

Figure S12: Extinction coefficient for hydrazone product **9** in sodium phosphate buffer 100 mM pH 7.5 at 287 nm: 19.2 $mM^{-1}cm^{-1}$. **(A)** UV-Vis absorbance spectra of hydrazone product **9** at different concentrations. **(B)** Absorbance at 287 nm of hydrazone product **10** at different concentrations.

Using Lambert-Beer law:

$$A = \varepsilon l C$$

, where A is the absorbance of **9** at 287 nm, ε is the extinction coefficient, l is the path length and C is the concentration of hydrazone **9**, εl is the slope of plot in Figure S12B. Therefore, extinction coefficients for hydrazone **9** at 287 nm is 19.2 mM^{-1} cm^{-1} at pH 7.5.

The yield of hydrazone product **9** is further calculated by determining the concentration from:

$$[C] = \frac{A}{\varepsilon l}$$

By monitoring the absorbance of product **9** peak at 287 nm, the yield of **9** as a function of time can be calculated. All graphs showing the yield of **9** in this work are calculated in this way.

7 Kinetic model

In this section the kinetic model is explained, which calculates the changing reaction rate constant for hydrazone formation during the cycle. When the esters are added to the reaction mixture, the catalyst is liberated from the CB[7] host and over time due to the hydrolysis of the esters the catalyst is captured again inside CB[7]. Consequently, the free catalyst concentration in the solution is changing as a function of time (and the catalytic rate constant). Overall, this can be modelled numerically with a set of differential equations. To do this, we will first explain the ester hydrolysis kinetics, hydrazone formation kinetics and equilibrium concentration calculations for host-guest complex formation. After that we assemble the kinetic model based on these individual parts and numerically calculate the concentration of the different species and the k-value.

7.1 Ester hydrolysis reaction kinetics

It is assumed that ester hydrolysis proceeds in a pseudo-first order rate:

$$Ester + Water \rightarrow Acid + Alcohol$$

$$\frac{d[E]}{dt} = -k_{hydrolysis}[E]$$

, where $k_{hydrolysis}$ is the hydrolysis rate constant and $[E]$ the time-dependent ester concentration. After integration, the rate law can be written as:

$$ln\left(\frac{[E]_t}{[E]_0}\right) = -k_{hydrolysis}t$$

, where $[E]_0$ is the initial ester concentration, $[E]_t$ the ester concentration at time t and $k_{hydrolysis}$ the hydrolysis rate constant. By following the ester vs acid concentration over time with ^{1}H NMR the hydrolysis rate constant can be determined by fitting the equation to a linear line (y = mx). The as such determined rate constants for ester hydrolysis at different pH and inside or outside CB[7] are provided in Figure S6-7.

7.2 Hydrazone formation reaction kinetics

The hydrazone formation reaction is assumed to be a second-order reaction. The reaction was performed at pseudo-first order conditions by using one of the reagents in excess. Unless stated otherwise, concentrations used in the hydrazone formation reaction were 0.2 mM aldehyde **7**, 0.02 mM hydrazide **8**.

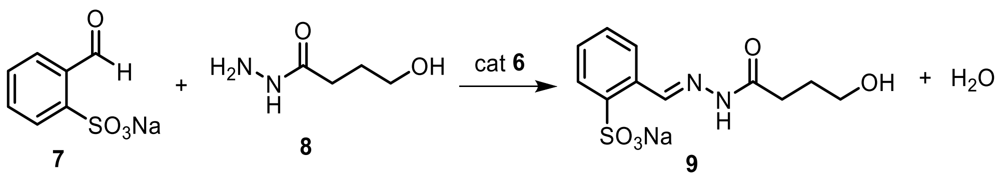

**Initial**	**t = 0**	$[A]_0$	$[A]_0$	0
	**t = *t***	$[A]_t = [A]_0 - [C]_t$	$[B]_t = [B]_0 - [C]_t$	$[C]_t$

$[A]$ is the excess aldehyde **7** concentration, $[B]$ the concentration of hydrazide **8** and $[C]$ the hydrazone product **9**. The second order rate equation, assuming first-order in both A and B, can be expressed as:

$$\frac{d[C]}{dt} = k[A][B]$$

For $[A]_0 \neq [B]_0$ and using $[A]_t = [A]_0 - [C]_t$ and $[B]_t = [B]_0 - [C]_t$ the second-order rate equation after integration can be expressed as:

$$\ln\frac{[A]_t}{[A]_0} - ln\frac{[B]_t}{[B]_0} = k([A]_0 - [B]_0)t$$

Because A was used in excess, it can be assumed that:

$[A]_0 \gg [B]_0$ then $\frac{[A]_t}{[A]_0} \approx 1$ and $[A]_0 - [B]_0 \approx [A]_0$

Therefore, the rate equation can be written as:

$$\frac{[B]_t}{[B]_0} = e^{-k[A]_0 t}$$

$$ln\left(\frac{[B]_t}{[B]_0}\right) = -k[A]_0 t$$

$$ln\left(1 - \frac{[C]_t}{[B]_0}\right) = -k[A]_0 t$$

, where $[A]_0$ is the initial aldehyde **7** concentration, $[B]_0$ the initial hydrazide **8** concentration, $[C]_t$ the hydrazone **9** concentration at time t and k the reaction rate constant. Hence, by following the hydrazone product **9** concentration over time with UV-VIS the rate constants of the pseudo-first order reactions can be determined by fitting this equation to a linear line (y = mx).

7.3 Equilibrium concentration calculation of dye/catalyst/ester⊂CB[7] complex

The calculation here is shown for the catalyst⊂CB[7] complex, but the same calculation applies for the dye⊂CB[7] and ester⊂CB[7] complex. The equilibrium concentrations can be calculated as follows:

	$[CB[7]]$	$[Cat]$	$[Cat \subset CB[7]]$
**Initial**	$[CB[7]_0] = a$	$[Cat]_0 = b$	0
**Change**	$-x$	$-x$	x
**Equilibrium**	$a - x$	$b - x$	x

, where $[CB[7]_0]$and $[Cat_0]$ are the CB[7] and catalyst concentrations that are added to the reaction mixture and x is the catalyst⊂CB[7] complex concentration. From this, the association constant can be defined as:

$$K_a = \frac{k_f}{k_b} = \frac{[Cat\subset CB[7]]}{[Cat][CB[7]]} = \frac{x}{(a-x)(b-x)}$$

By solving this equation for x an expression for the catalyst⊂CB[7] complex concentration is obtained:

$$[Cat\subset CB[7]] = \frac{a \cdot K_a + b \cdot K_a + 1 - \sqrt{a^2 \cdot K_a{}^2 - 2a \cdot b \cdot K_a{}^2 + b^2 \cdot K_a{}^2 + 2 \cdot a \cdot K_a + 2 \cdot b \cdot K_a + 1}}{2 \cdot K_a}$$

$$= \frac{[CB[7]]_0 K_a + [Cat]_0 K_a + 1 - \sqrt{[CB[7]]_0{}^2 K_a{}^2 - 2[CB[7]]_0 [Cat]_0 K_a{}^2 + [Cat]_0{}^2 K_a{}^2 + 2[CB[7]]_0 K_a + 2[Cat]_0 K_a + 1}}{2K_a}$$

From this, the free CB[7] and catalyst concentrations in solution can then be calculated as follows:

$$[CB[7]] = [CB[7]]_0 - [Cat\subset CB[7]]$$

$$[Cat] = [Cat]_0 - [Cat\subset CB[7]]$$

, where $[CB[7]]$ is the uncomplexed (free) CB[7] concentration and $[Cat]$ is the uncomplexed (free) catalyst concentration.

7.4 Matlab numerical model of differential equations

Using the above relations and by determining the ester hydrolysis rate constants in and outside CB[7] with ^{1}H NMR, the catalyst in the solution as a function of time can be calculated and based on that the rate constant for hydrazone formation over time. Upon addition of the esters, the esters will form a complex with CB[7] with a concentration of $[Ester\subset \mathrm{CB[7]}](t)$, which is a function of time (as the esters hydrolyse over time). The ester inside CB[7] hydrolyses slower than outside CB[7] this is taken into account in the model. Hence, to calculate the concentration profiles of all the species in the reaction mixture, we use the following equations and use the Matlab function ode45 to calculate them numerically:

$$\frac{d[Ester]}{dt} = -k_{hydrolysis}[Ester] - k_f[E][CB[7]] + k_b[Ester\subset CB[7]]$$

$$\frac{d[Ester\subset CB[7]]}{dt} = -k_{hydrolysis}[Ester\subset \mathrm{CB[7]}] + k_f[Ester][CB[7]] - k_b[Ester\subset CB[7]]$$

$$\frac{d[Acid]}{dt} = k_{hydrolysis}[Ester] + k_{hydrolysis}[Ester\subset CB[7]]$$

$$\frac{d[Alcohol]}{dt} = k_{hydrolysis}[Ester] + k_{hydrolysis}[Ester\subset CB[7]]$$

$$\frac{d[Cat]}{dt} = -k_f[Cat][CB[7]] + k_b[Cat\subset CB[7]]$$

$$\frac{d[Cat\subset CB[7]]}{dt} = k_f[Cat][CB[7]] - k_b[Cat\subset CB[7]]$$

$$\frac{d[CB]}{dt} = -k_f[Ester][CB[7]] + k_b[Ester\subset CB[7]] - k_f[Cat][CB[7]] + k_b[Cat\subset CB[7]] + k_{hydrolysis}[Ester\subset CB[7]]$$

Finally, to calculate the rate constant as a function of time the catalyst concentration over time is used in the following equation:

$$k\,(t) = (k_0 + k_{cat}[cat](t) + k_1[CB[7]])$$

, where $k\,(t)$ is the total reaction rate constant that changes over time, k_0 the rate constant of the background reaction (uncatalysed hydrazone formation), k_1 the rate constant of CB[7] catalyzed hydrazone formation, $[cat](t)$ the catalyst concentration over time and $[CB[7]]$ the total CB[7] concentration present. N.B. CB[7] has a minor background reaction taken into account with $k_1[CB[7]]$.

8 Hydrazone formation reactions

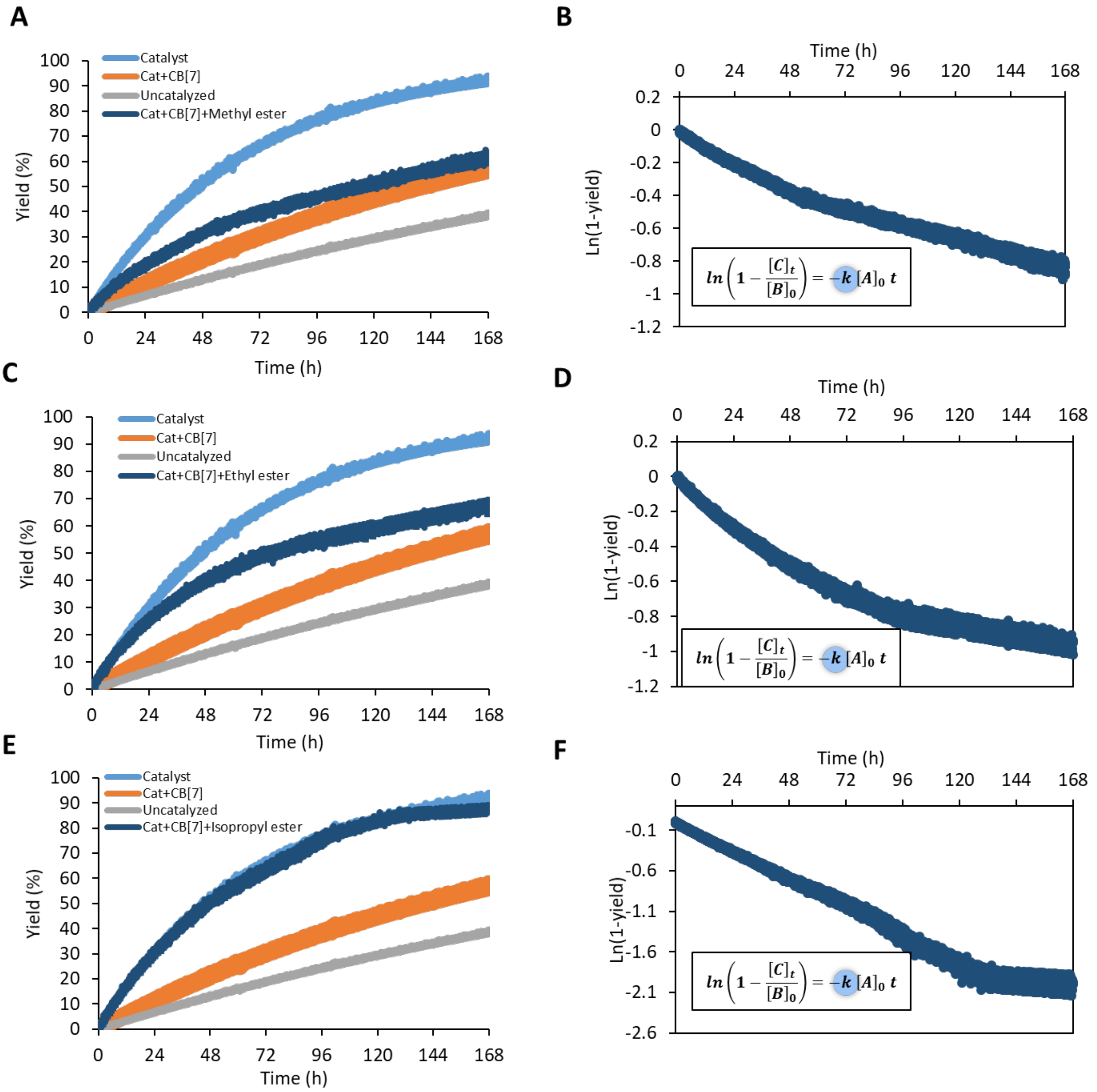

Figure S13: Yield of hydrazone **9 (A, C and E)** for the different esters and the calculated slope for the ester experiments (B, D and F): **(A-B)** Methyl ester **1**, **(C-D)** Ethyl ester **2** and **(E-F)** Isopropyl ester **3**. Conditions: 0.2 mM aldehyde **7**, 0.02 mM hydrazide **8**, 0.2 mM catalyst **6**, 0.6 mM CB[7] and 2.5 mM methyl ester **1** / 1.5 mM ethyl ester **2** / 0.8 mM isopropyl ester **3** in sodium phosphate buffer 100 mM, pH 7.5 at RT.

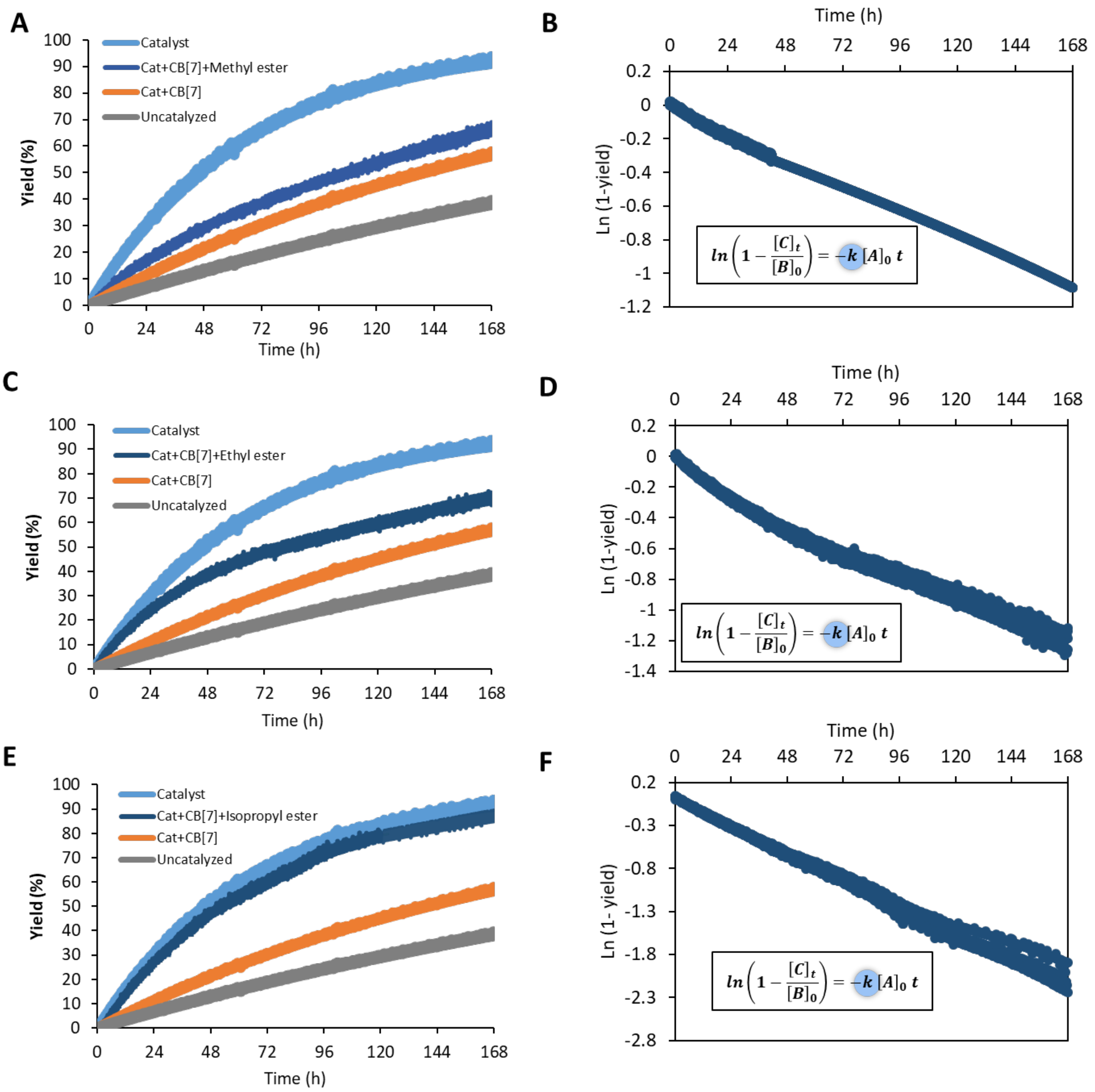

Figure S14: Yield of hydrazone **9 (A, C and E)** for the different esters and the calculated slope for the ester experiments (B, D and F): **(A-B)** Methyl ester **1**, **(C-D)** Ethyl ester **2** and **(E-F)** Isopropyl ester **3**. Conditions: 0.2 mM aldehyde **7**, 0.02 mM hydrazide **8**, 0.2 mM catalyst **6**, 0.6 mM CB[7] and 2 mM methyl ester **1** / 1 mM ethyl ester **2** / 0.75 mM isopropyl ester **3** in sodium phosphate buffer 100 mM, pH 7.5 at RT.

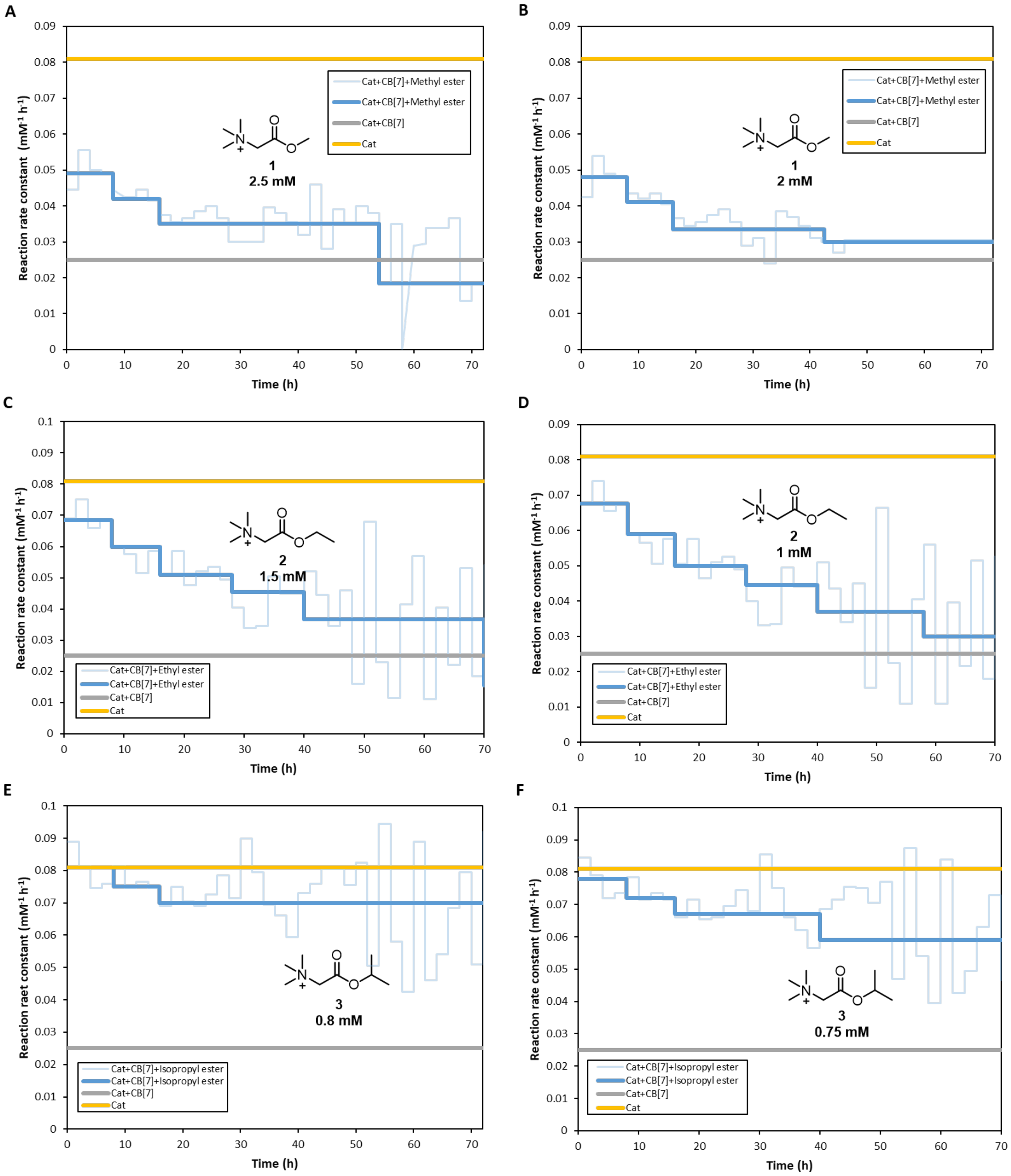

Figure S15: Rate constant for hydrazone formation as a function of time at various catalytic conditions. Experimental data with esters at small (blue transparent lines) and large time intervals (blue thick lines): **(A)** Methyl ester **1** 2.5 mM, **(B)** Methyl ester **1** 2 mM, **(C)** Ethyl ester **2** 1.5 mM, **(D)** Ethyl ester **2** 1 mM, **(E)** Isopropyl ester **3** 0.8 mM, **(F)** Isopropyl ester **3** 0.75 mM. Conditions: 0.2 mM aldehyde **7**, 0.02 mM hydrazide **8**, 0.2 mM catalyst **6** and 0.6 mM CB[7].

Table S2. Overview of the reaction rates and R^2 at selected time periods referring to Figures 3 and 4 in the manuscript.

Conditions: 2.5 mM Methyl ester **1**, 0.2 mM aldehyde **7**, 0.02 mM hydrazide **8**, 0.2 mM catalyst **6** and 0.6 mM CB[7] in pH 7.5, 100 mM sodium phosphate buffer solution (Figure 3E).

**Time period (h)**	***k*** **(mM^{-1} h^{-1})**	**R^2**
0-8	0.049	0.9460
8-16	0.042	0.9082
16-54	0.035	0.9871
54-168	0.018	0.9721

Conditions: 2 mM Methyl ester **1**, 0.2 mM aldehyde **7**, 0.02 mM hydrazide **8**, 0.2 mM catalyst **6** and 0.6 mM CB[7] in pH 7.5, 100 mM sodium phosphate buffer solution (Figure 3H).

**Time period (h)**	***k*** **(mM^{-1} h^{-1})**	**R^2**
0-8	0.048	0.9456
8-16	0.041	0.9082
16-42.5	0.034	0.9789
42.5-168	0.003	0.9988

Conditions: 1.5 mM Ethyl ester **2**, 0.2 mM aldehyde **7**, 0.02 mM hydrazide **8**, 0.2 mM catalyst **6** and 0.6 mM CB[7] in pH 7.5, 100 mM sodium phosphate buffer solution (Figure 3F).

**Time period (h)**	***k*** **(mM^{-1} h^{-1})**	**R^2**
0-8	0.069	0.966
8-16	0.060	0.9445
16-28	0.051	0.9495
28-40	0.046	0.9171
40-70	0.037	0.9598
70-168	0.016	0.8853

Conditions: 1 mM Ethyl ester **2**, 0.2 mM aldehyde **7**, 0.02 mM hydrazide **8**, 0.2 mM catalyst **6** and 0.6 mM CB[7] in pH 7.5, 100 mM sodium phosphate buffer solution (Figure 3I).

**Time period (h)**	***k*** **(mM^{-1} h^{-1})**	**R^2**
0-8	0.068	0.966
8-16	0.059	0.9445
16-28	0.050	0.9495
28-40	0.045	0.9171
40-58	0.037	0.9172
58-82	0.030	0.8563
82-168	0.029	0.9443

Conditions: 0.8 mM Isopropyl ester **3**, 0.2 mM aldehyde **7**, 0.02 mM hydrazide **8**, 0.2 mM catalyst **6** and 0.6 mM CB[7] in pH 7.5, 100 mM sodium phosphate buffer solution (Figure 3G).

**Time period (h)**	***k*** **(mM^{-1} h^{-1})**	**R^2**
0-8	0.081	0.9764
8-16	0.075	0.9589
16-138	0.070	0.9923
138-168	0.011	0.0059

Conditions: 0.75 mM Isopropyl ester **3**, 0.2 mM aldehyde **7**, 0.02 mM hydrazide **8**, 0.2 mM catalyst **6** and 0.6 mM CB[7] in pH 7.5, 100 mM sodium phosphate buffer solution (Figure 3J).

**Time period (h)**	***k*** **(mM^{-1} h^{-1})**	**R^2**	**Note**
0-8	0.078	0.9757	

8-16	0.072	0.9589	
16-40	0.067	0.9885	
40-124	0.059	0.9832	
124-168	0.048	0.2967	

Conditions: 0.2 mM aldehyde **7**, 0.02 mM hydrazide **8**, 0.2 mM catalyst **6** and 0.6 mM CB[7] in pH 7.5, 100 mM sodium phosphate buffer solution, 2.5 mM Methyl ester **1** was added at t = 8 h (Figure 4B).

**Time period (h)**	***k*** **(mM^{-1} h^{-1})**	**R^2**	**Note**
0-8	0.024	0.8118	No ester added
8-12	0.051	0.823	**1** added
12-20	0.048	0.9339	
20-30	0.039	0.9108	
30-55	0.036	0.9738	
55-70	0.028	0.6973	
70-168	0.020	0.9644	

Conditions: 0.2 mM aldehyde **7**, 0.02 mM hydrazide **8**, 0.2 mM catalyst **6** and 0.6 mM CB[7] in pH 7.5, 100 mM sodium phosphate buffer solution, 1.5 mM Ethyl ester **2** was added at t = 8 h (Figure 4C).

**Time period (h)**	***k*** **(mM^{-1} h^{-1})**	**R^2**	**Note**
0-8	0.024	0.8119	No ester added
8-18	0.069	0.9757	**2** added
18-30	0.058	0.9655	
30-40	0.051	0.9130	
40-50	0.047	0.8683	
50-80	0.039	0.9564	
80-100	0.030	0.7911	

Conditions: 0.2 mM aldehyde **7**, 0.02 mM hydrazide **8**, 0.2 mM catalyst **6** and 0.6 mM CB[7] in pH 7.5, 100 mM sodium phosphate buffer solution, 0.8 mM Isopropyl ester **3** was added at t = 8 h (Figure 4D).

**Time period (h)**	***k*** **(mM^{-1} h^{-1})**	**R^2**	**Note**
0-8	0.024	0.8115	No ester added
8-40	0.077	0.9959	**3** added
40-60	0.075	0.9698	
60-80	0.068	0.9222	
80-130	0.068	0.9485	
130-176	0.023	0.2648	

Conditions: 0.2 mM aldehyde **7**, 0.02 mM hydrazide **8**, 0.2 mM catalyst **6** and 0.42 mM CB[7] in pH 7.6, 100 mM sodium phosphate buffer solution, 2.5 mM Methyl ester **1** was added at t = 8, 32, 56 h (Figure 4F).

**Time period (h)**	***k*** **(mM^{-1} h^{-1})**	**R^2**	**note**
0-8	0.020	0.9968	No ester added
8-16	0.047	0.9985	
16-24	0.037	0.9984	first addition
24-32	0.032	0.9969	
32-40	0.053	0.9980	
40-48	0.043	0.9974	second addition
48-56	0.033	0.9953	
56-64	0.051	0.9946	
64-72	0.044	0.9965	third addition
72-80	0.035	0.9927	

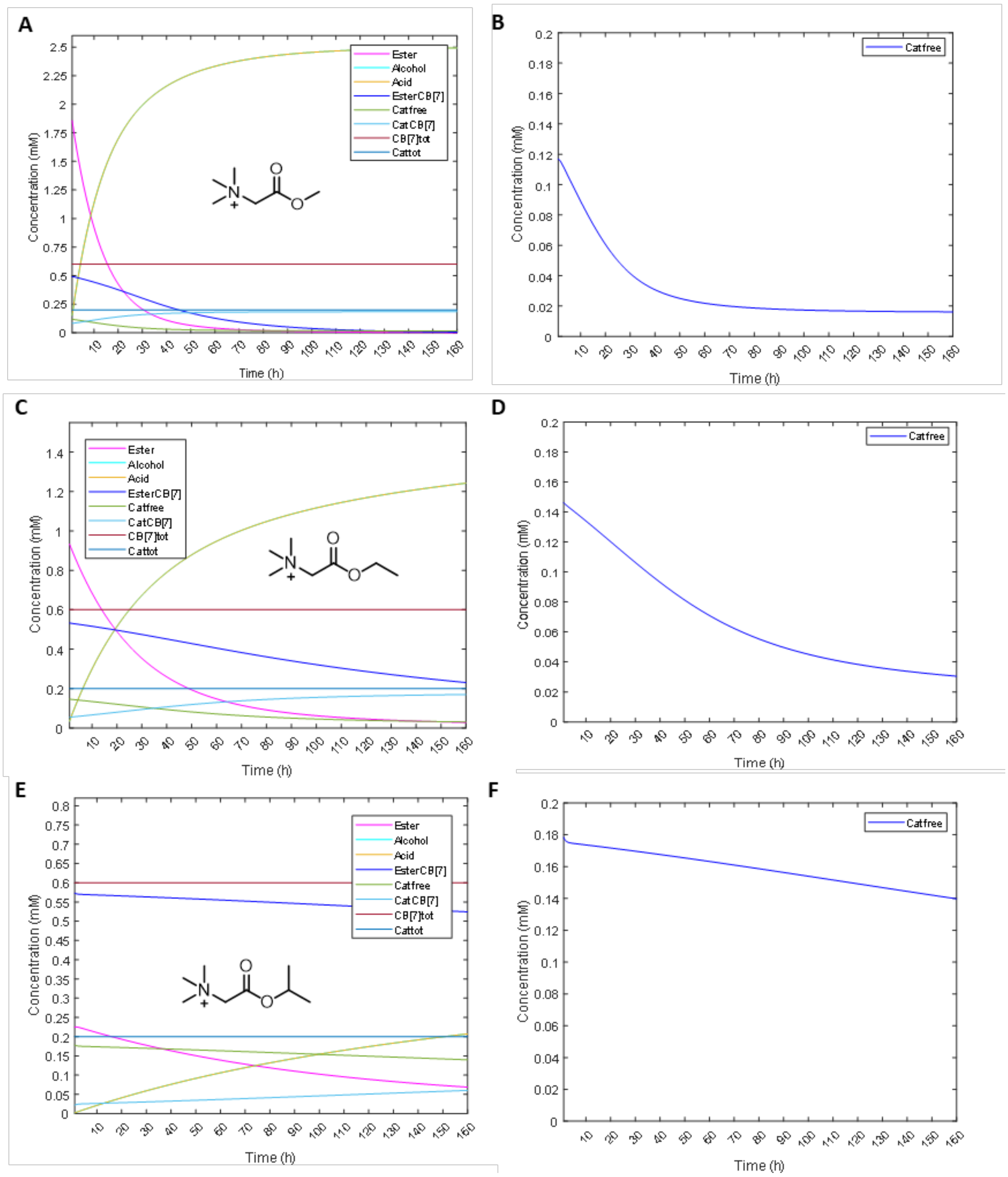

Figure S16: Concentration profiles of all species (A, C and E) and the free catalyst (B, D and F) over time calculated with the kinetic model in Matlab: **(A-B)** Methyl ester **1** 2.5 mM, **(C-D)** Ethyl ester **2** 1.5 mM and **(E-F)** Isopropyl ester **3** 0.8 mM.

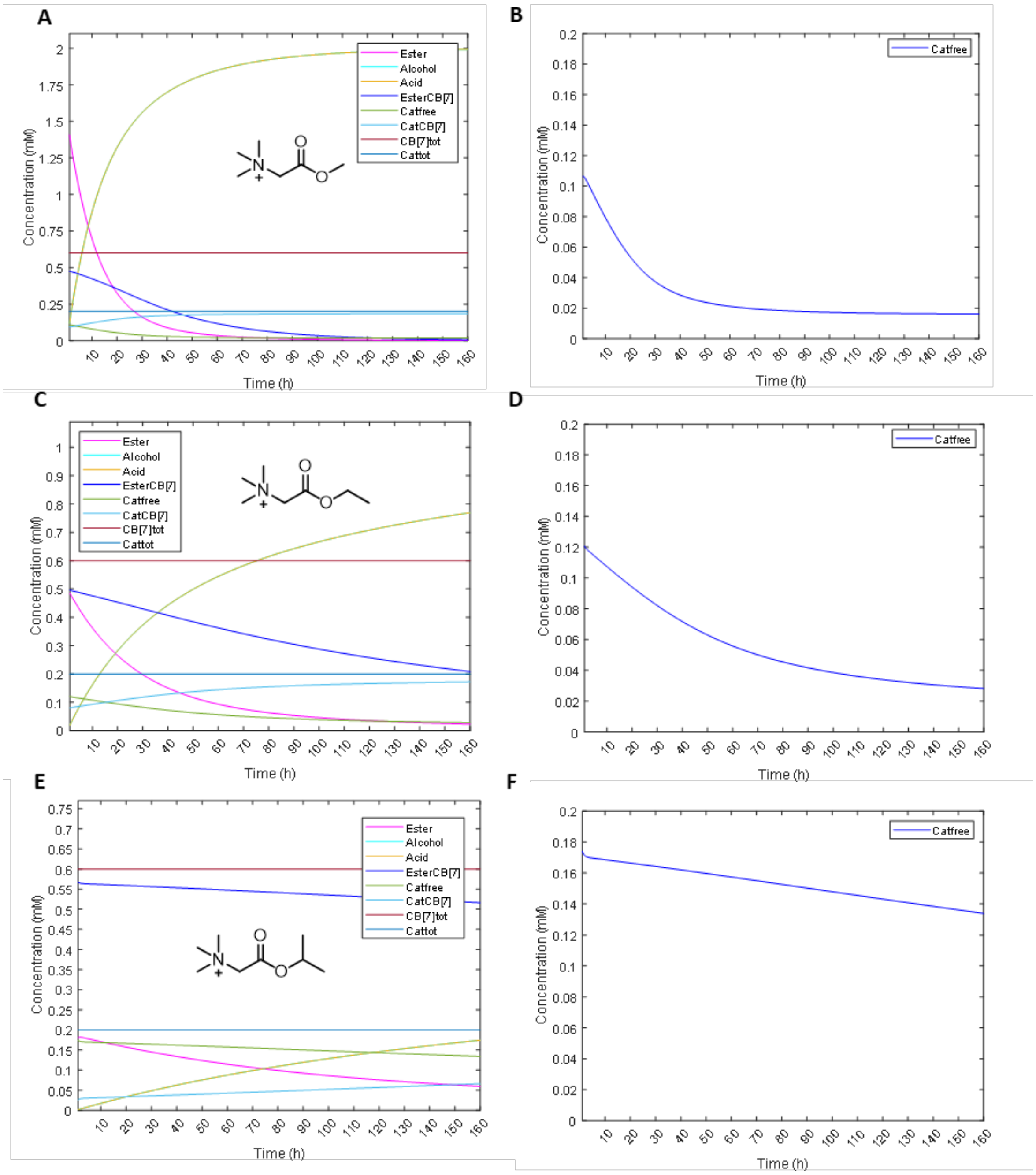

Figure S17: Concentration profiles of all species (A, C and E) and the free catalyst (B, D and F) over time calculated with the kinetic model in Matlab: **(A-B)** Methyl ester **1** 2 mM, **(C-D)** Ethyl ester **2** 1 mM and **(E-F)** Isopropyl ester **3** 0.75 mM.

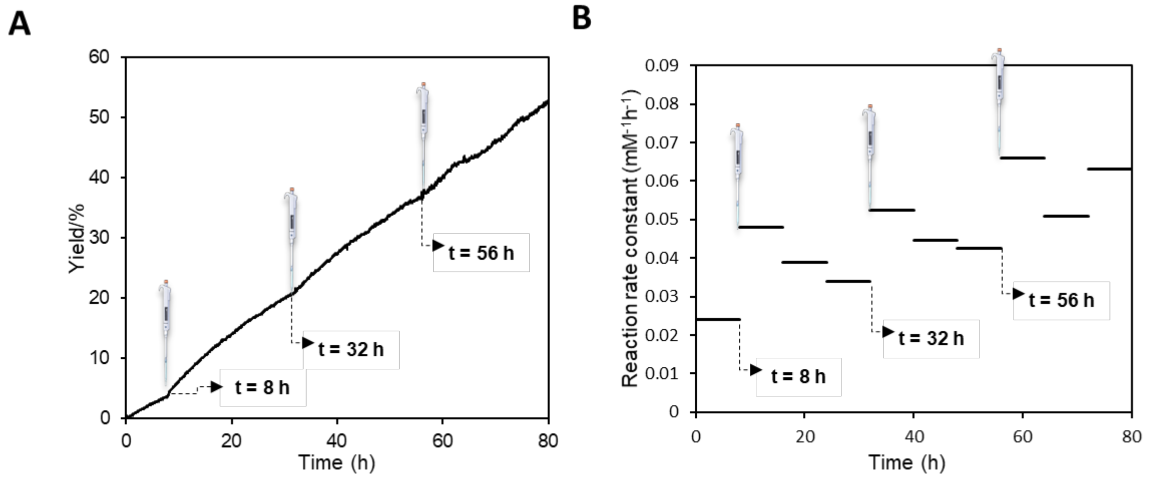

Figure S18: (A) Yield of hydrazone **9** for 3 times addition of methyl ester **1** at t= 8h, 32 h and 56 h. **(B)** Reaction rate constant for hydrazone **9** formation as a function of time. Conditions: 2.5 mM methyl ester **1** (×3), 0.2 mM aldehyde **7**, 0.02 mM hydrazide **8**, 0.2 mM catalyst **6** and 0.6 mM CB[7] in pH 7.5, 100 mM sodium phosphate buffer solution.

9 Control experiments

9.1 Ester blank reactions

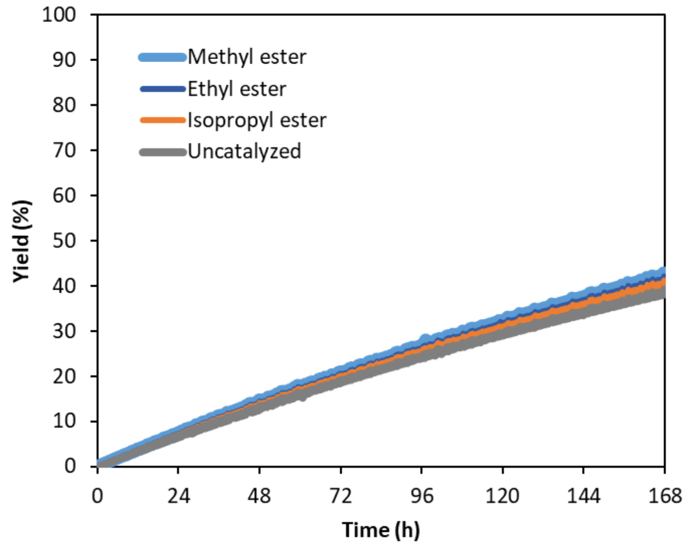

Catalyst system	Second order k_r [mM^{-1} h^{-1}]	$k_i/k_{uncatalyzed}$
Uncatalyzed	0.0135	1
Methyl ester	0.0140	1.04
Ethyl ester	0.0137	1.01
Isopropyl ester	0.0136	1.01

Figure S1**9**: Hydrazone formation blank reactions with esters **1-3**. Conditions: 0.2 mM aldehyde **7**, 0.02 mM hydrazide **8** with methyl ester **1** 2.5 mM or ethyl ester **2** 1.5 mM or isopropyl ester **3** 0.8 mM in sodium phosphate buffer 100 mM, pH 7.5 at RT. Very similar trend to the uncatalyzed reaction of yield vs time indicates that the esters do not catalyze the hydrazone formation reaction.

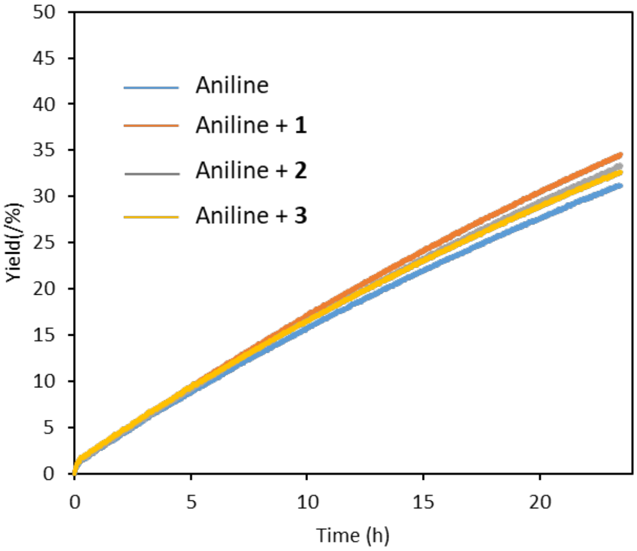

Catalyst system	Reaction rate k_r ($mM^{-1} h^{-1}$)	$k_i/k_{aniline}$
Aniline	0.078	1
Aniline + **1**	0.088	1.14
Aniline + **2**	0.084	1.08
Aniline + **3**	0.081	1.05

Figure S20: Hydrazone formation reactions with aniline by adding esters **1-3**. Conditions: 0.2 mM aldehyde **7**, 0.02 mM hydrazide **8** and 0.2 mM aniline **6**, with methyl ester **1** 2.5 mM or ethyl ester **2** 1.5 mM or isopropyl ester **3** 0.8 mM in sodium phosphate buffer 100 mM, pH 7.5 at RT. Addition of all esters increased the yield slightly.[5]

9.2 NMR and MS controls

A long-term ^{1}H NMR measurement was performed for the aniline catalysed hydrazone formation reaction in the presence of esters **1**-**3** to verify the formation of any possible side products (Conditions: 2 mM aldehyde **7**, 0.2 mM hydrazide **8** and 2 mM aniline **6,** with methyl ester **1** 25 mM, ethyl ester **2** 15 mM, isopropyl ester **3**, in pH 7.5, 100 mM sodium phosphate buffer solution and 10% D_2O). However, due to the large difference in concentrations of reactants, catalysts and esters, the peaks cannot be visualized in one scale. Therefore, two scales of the same spectrum have been provided in Figure S21-S23 to show each peak.

On top of that, LC-MS of these reaction solutions after 8 days were also measured, however no identifiable side products were observed.

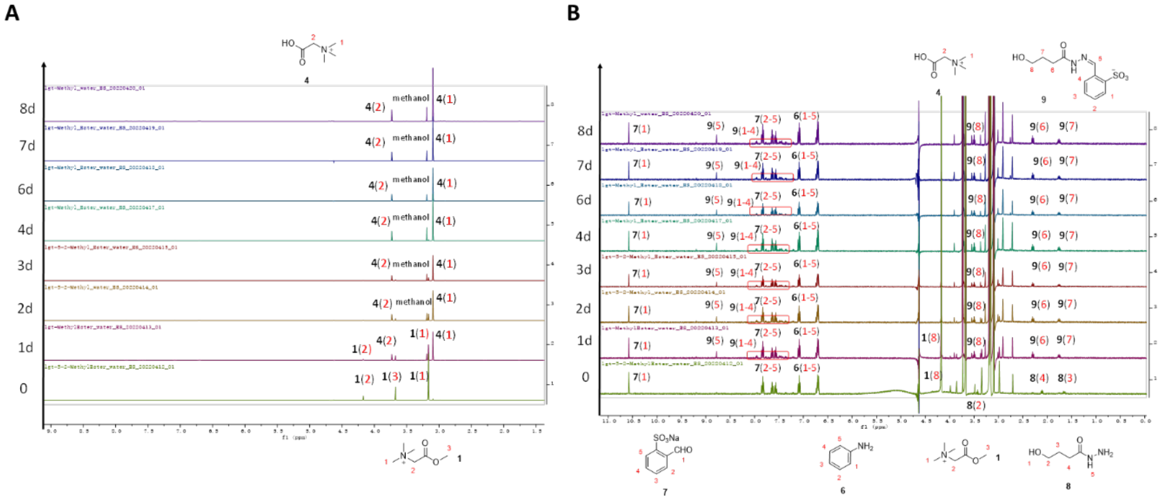

Figure S21: Stacked NMR spectra of hydrazone **9** formation reaction catalyzed by aniline after adding ester **1**: **(B)** is a zoom in of **(A)**. Conditions: 2 mM aldehyde **7**, 0.2 mM hydrazide **8** and 2 mM aniline **6**, with methyl ester **1** 25 mM, in pH 7.5, 100 mM sodium phosphate buffer solution and 10% D_2O. The red boxes in (**B**) refer to the aromatic protons of **9**.

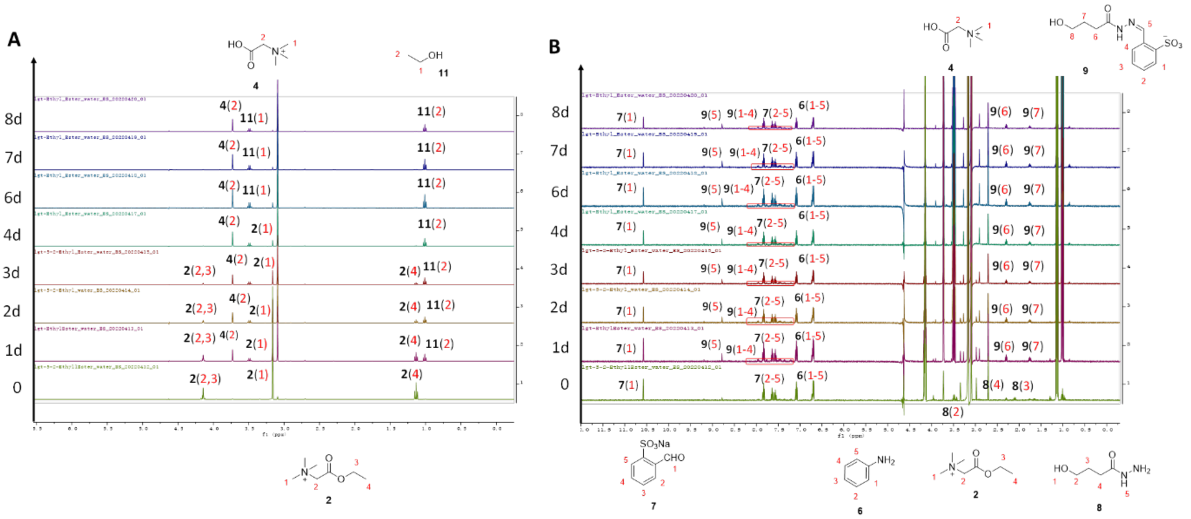

Figure S22: Stacked NMR spectra of hydrazone **9** formation reaction catalyzed by aniline after adding ester **2**: **(B)** is a zoom in of **(A)**. Conditions: 2 mM aldehyde **7**, 0.2 mM hydrazide **8** and 2 mM aniline **6**, with ethyl ester **2** 15 mM, in pH 7.5, 100 mM sodium phosphate buffer solution and 10% D_2O. The red boxes in (**B**) refer to the aromatic protons of **9**.

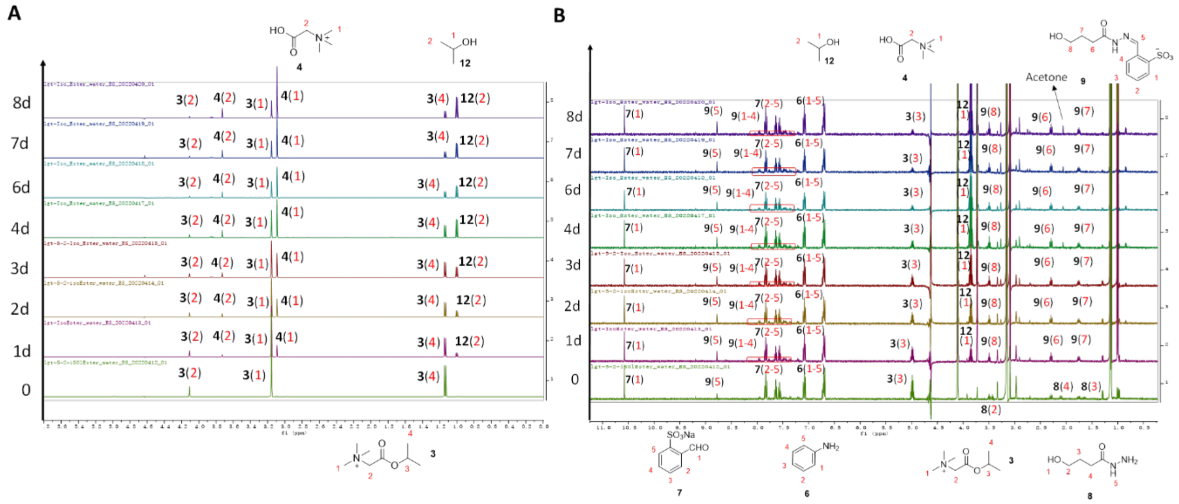

Figure S23: Stacked NMR spectra of hydrazone **9** formation reaction catalyzed by aniline after adding ester **3**: **(B)** is a zoom in of **(A)**. Conditions: 2 mM aldehyde **7**, 0.2 mM hydrazide **8** and 2 mM aniline **6**, with isopropyl ester **3** 8 mM, in pH 7.5, 100 mM sodium phosphate buffer solution and 10% D_2O. The red boxes in (**B**) refer to the aromatic protons of **9**.

10 NMR and MS spectra

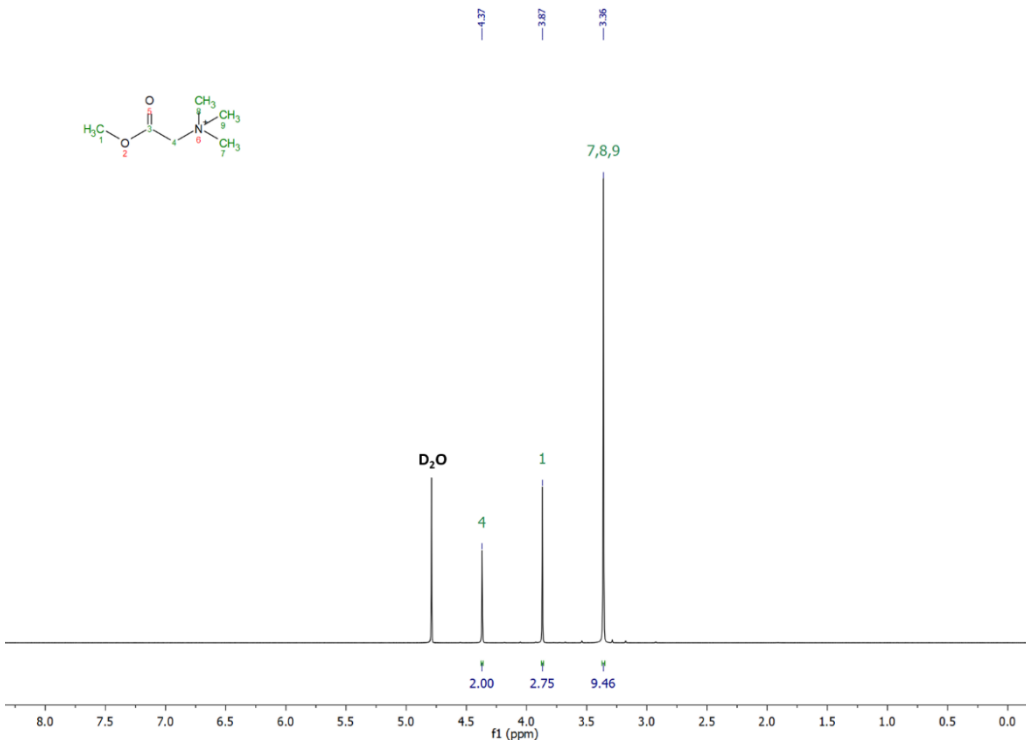

Figure S24: ^{1}H NMR spectrum of glycine betaine methyl ester **1** in D_2O.

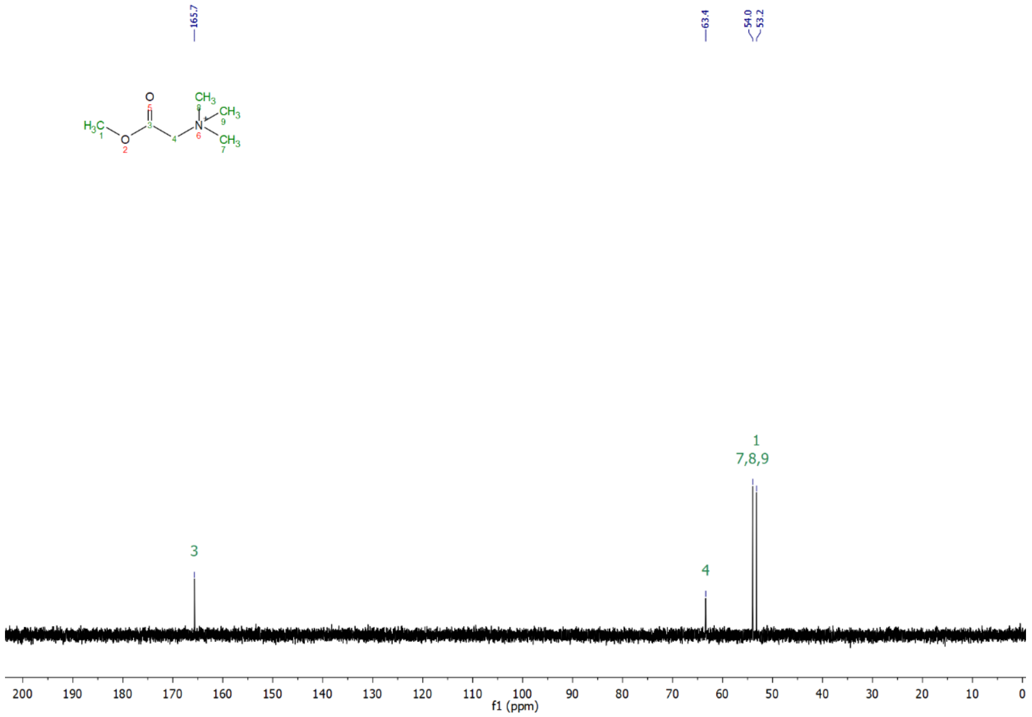

Figure S25: ^{13}C NMR spectrum of glycine betaine methyl ester **1** in D_2O.

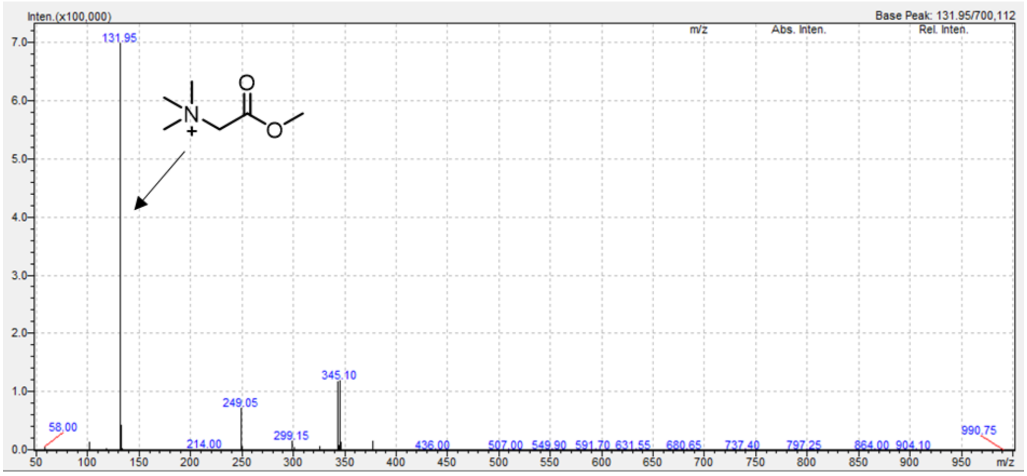

Figure S26: ESI-MS spectrum (positive mode) of glycine betaine methyl ester **1** in H_2O. m/z 132 is [(M)$^+$] and 343/ 345 is [(2M$^+$+Br$^-$)$^+$].

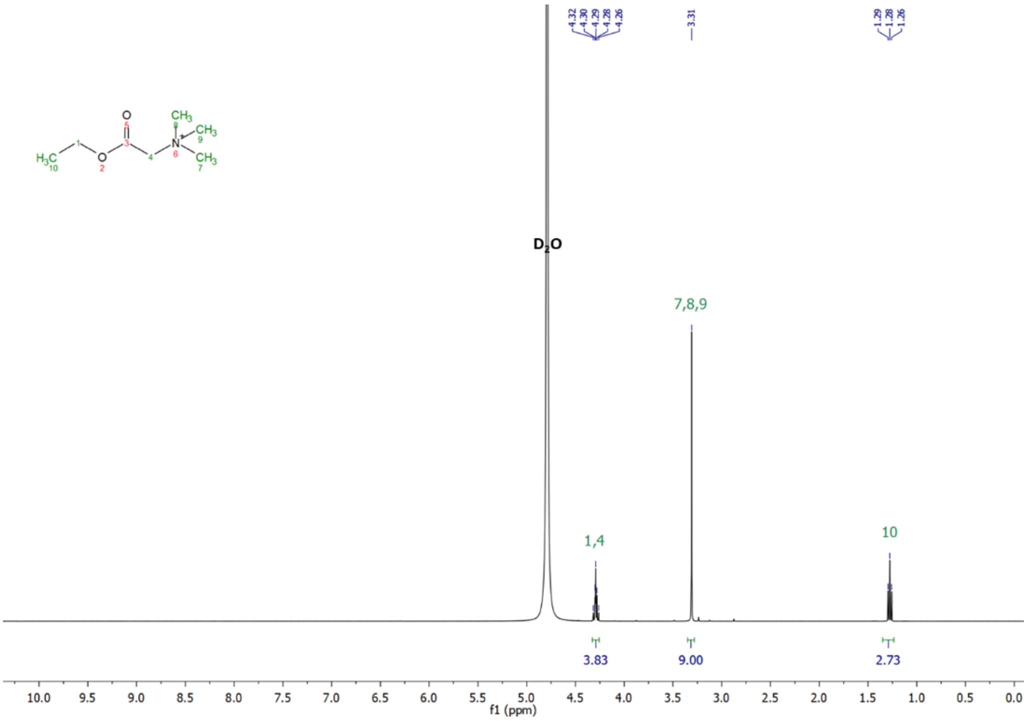

Figure S27: ^{1}H NMR spectrum of glycine betaine ethyl ester **2** in D_2O.

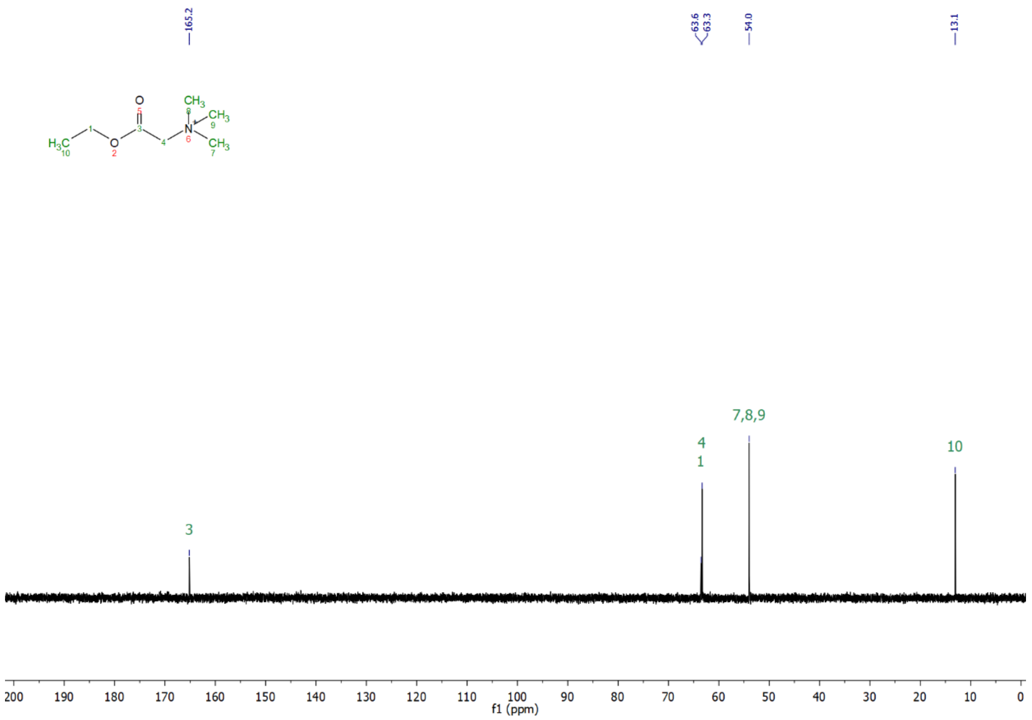

Figure S28: ^{13}C NMR spectrum of glycine betaine methyl ester **2** in D_2O.

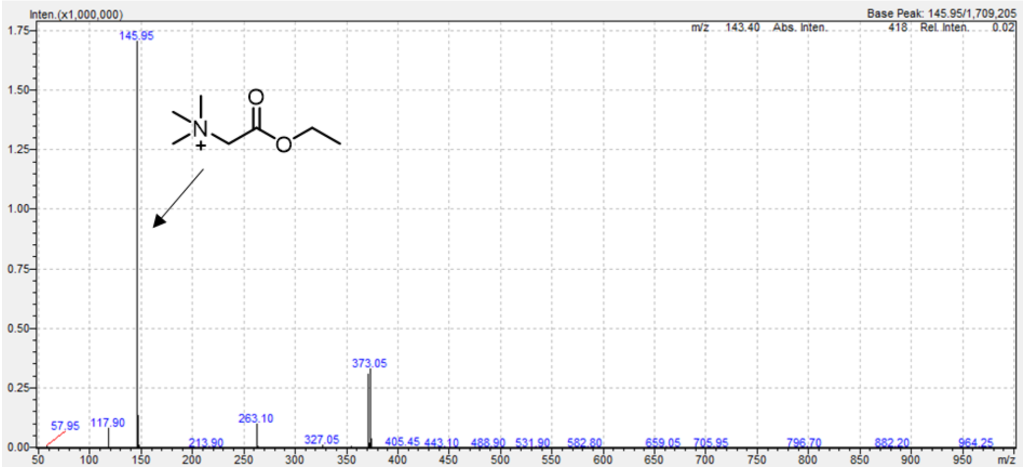

Figure S29: ESI-MS spectrum (positive mode) of glycine betaine ethyl ester **2** in H_2O. m/z 146 is $[(M)^+]$ and 371/ 373 is $[(2M^+ + Br^-)^+]$.

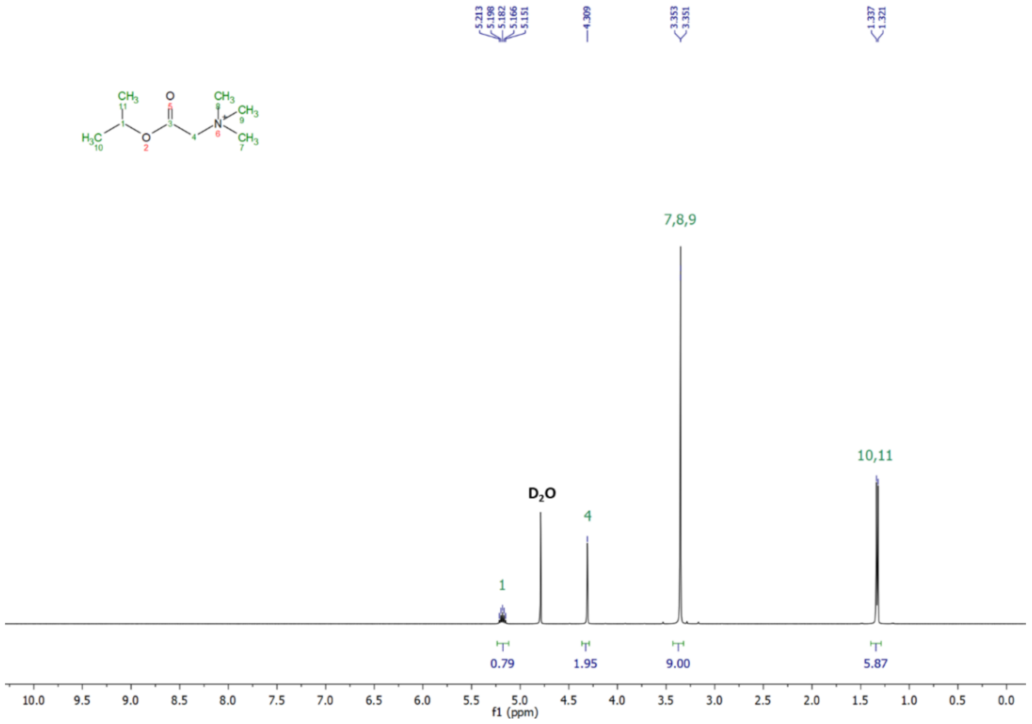

Figure S30: ^{1}H NMR spectrum of glycine betaine isopropyl ester **3** in D_2O.

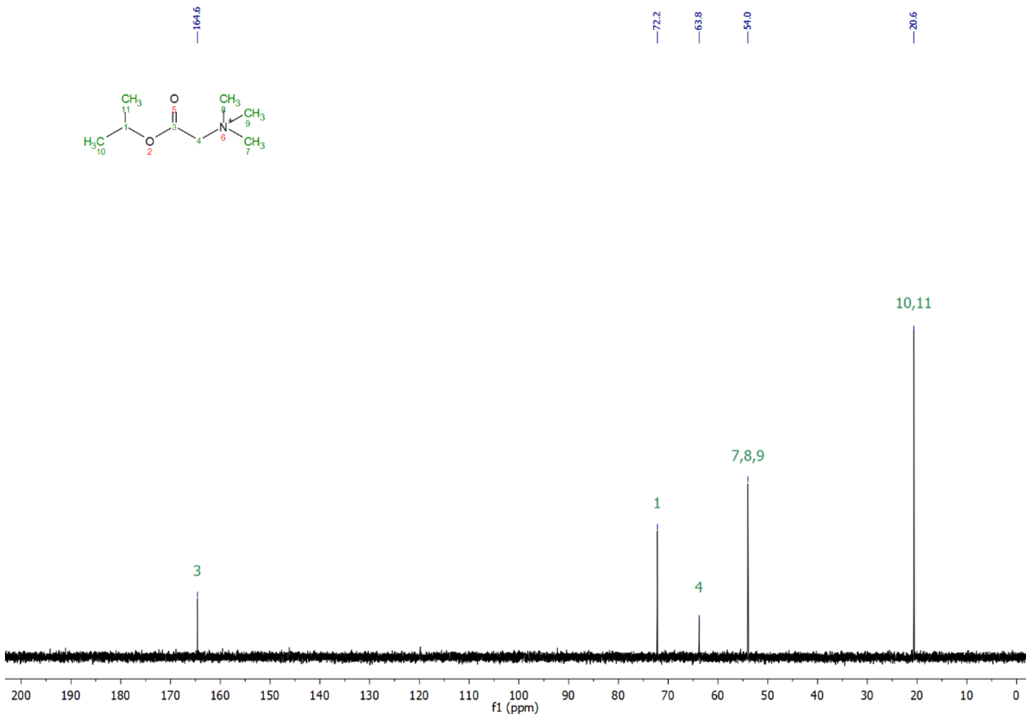

Figure S31: ^{13}C NMR spectrum of glycine betaine isopropyl ester **3** in D_2O.

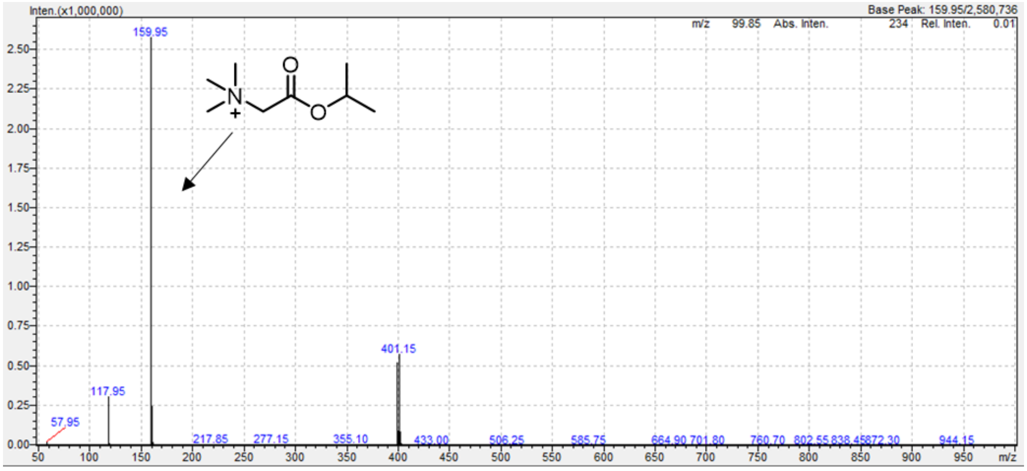

Figure S32: ESI-MS spectrum (positive mode) of glycine betaine isopropyl ester **3** in H_2O. m/z 160 is [$(M)^+$] and 399/ 401 is [$(2M^+ + Br^-)^+$].

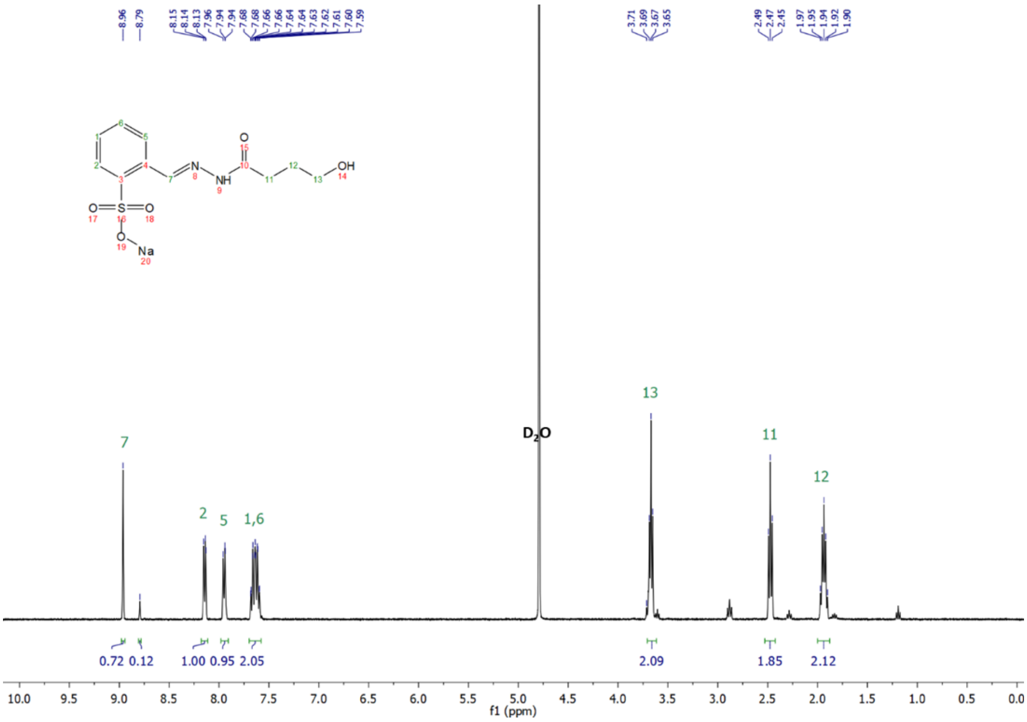

Figure S33: ^{1}H NMR spectrum of hydrazone product **9** in D_2O.

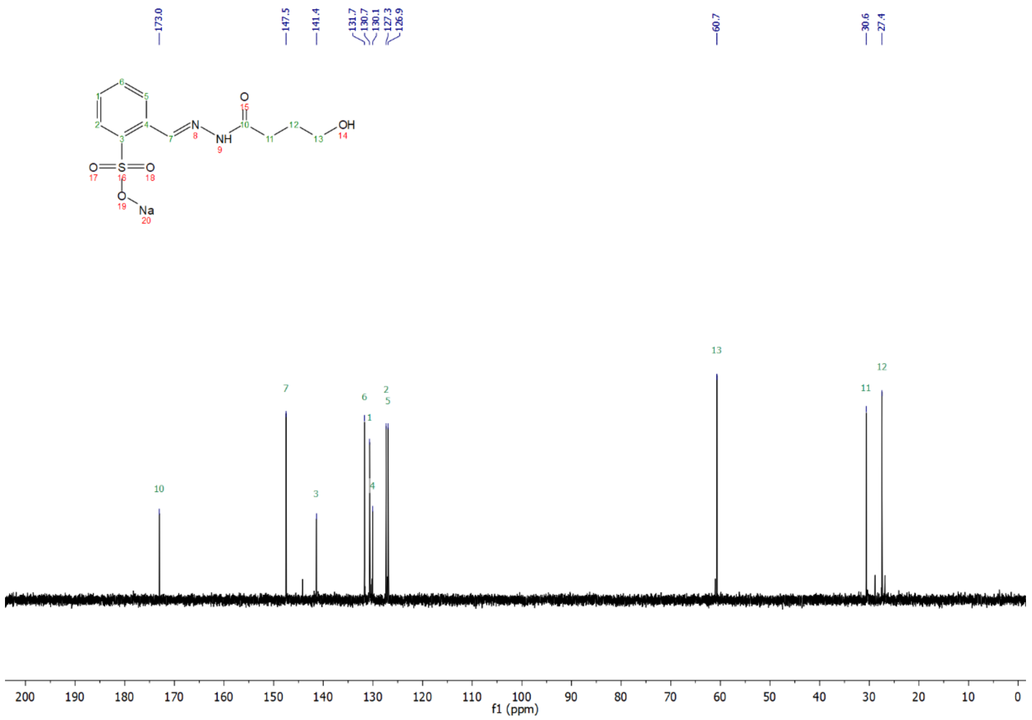

Figure S34: ^{13}C NMR spectrum of hydrazone product **9** in D_2O.

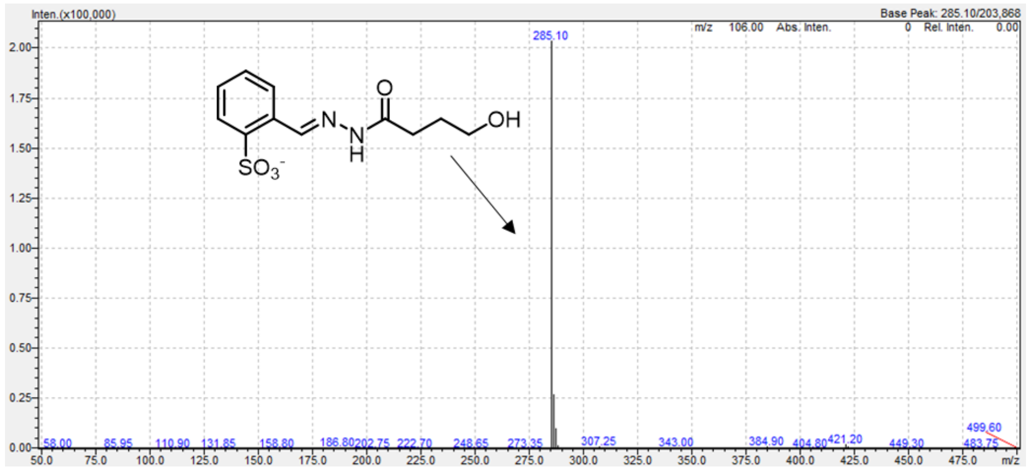

Figure S35: ESI-MS spectrum (negative mode) of hydrazone product **9** in H_2O. m/z 285 is $[(M-Na^+)^-]$.

References

1. Novacek, J., et al., *Towards a General Understanding of Carbonyl-Stabilised Ammonium Ylide-Mediated Epoxidation Reactions.* Chem. Eur. J., 2016. **22**, 11422-11428.
2. Li, G., et al., *Tuneable control over organocatalytic activity through host guest chemistry.* Angew. Chem. Int. Ed. 2021, DOI 10.1002/anie.202102227.
3. Shaikh, M., et al., *Complexation of acridine orange by cucurbit [7] uril and β-cyclodextrin: photophysical effects and pKa shifts.* Photochem. Photobiol. Sci., 2008. **7**, 408-414.
4. Liu, J., et al., *Insight into unusual downfield NMR shifts in the inclusion complex of acridine orange with cucurbit [7] uril.* Eur. J. Org. Chem. **2009**, 4931-4938.
5. Crisalli, P., Kool, E. T. Water-soluble organocatalysts for hydrazone and oxime formation. *J. Org. Chem.,* **2013**, *78*, 1184–1189.
